# The Olfactory Transcriptome and Progression of Sexual Maturation in Homing Chum Salmon *Oncorhynchus keta*

**DOI:** 10.1371/journal.pone.0137404

**Published:** 2015-09-23

**Authors:** Arjan P. Palstra, Kosuke Fukaya, Hiroaki Chiba, Ron P. Dirks, Josep V. Planas, Hiroshi Ueda

**Affiliations:** 1 Institute for Marine Resources and Ecosystem Studies (IMARES), Wageningen University and Research Centre, Korringaweg 5, 4401 NT Yerseke, The Netherlands; 2 Animal Breeding and Genomics Centre, Wageningen UR Livestock Research, PO Box 338, 6700 AH Wageningen, The Netherlands; 3 Laboratory of Aquatic Bioresources and Ecosystem, Section of Ecosystem Conservation, Field Science Center for Northern Biosphere, Hokkaido University, Sapporo, 060-0809 Hokkaido, Japan; 4 School of Marine Biosciences, Kitasato University, Kitasato 1-15-1, Minami-ku, Sagamihara, Kanagawa 252-0373, Japan; 5 ZF-screens BV, J.H. Oortweg 19, 2333 CH Leiden, The Netherlands; 6 Departament de Fisiologia i Immunologia, Facultat de Biologia, Universitat de Barcelona and Institut de Biomedicina de la Universitat de Barcelona (IBUB), Barcelona, Spain; University of Hyderabad, INDIA

## Abstract

Reproductive homing migration of salmonids requires accurate interaction between the reception of external olfactory cues for navigation to the spawning grounds and the regulation of sexual maturation processes. This study aimed at providing insights into the hypothesized functional link between olfactory sensing of the spawning ground and final sexual maturation. We have therefore assessed the presence and expression levels of olfactory genes by RNA sequencing (RNAseq) of the olfactory rosettes in homing chum salmon *Oncorhynchus keta* Walbaum from the coastal sea to 75 km upstream the rivers at the pre-spawning ground. The progression of sexual maturation along the brain-pituitary-gonadal axis was assessed through determination of plasma steroid levels by time-resolved fluoroimmunoassays (TR-FIA), pituitary gonadotropin subunit expression and *salmon gonadotropin-releasing hormone* (*sgnrh*) expression in the brain by quantitative real-time PCR. RNAseq revealed the expression of 75 known and 27 unknown salmonid olfactory genes of which 13 genes were differentially expressed between fish from the pre-spawning area and from the coastal area, suggesting an important role of these genes in homing. A clear progression towards final maturation was characterised by higher plasma 17α,20β-dihydroxy-4-pregnen-3-one (DHP) levels, increased pituitary *luteinizing hormone β subunit* (*lhβ*) expression and *sgnrh* expression in the post brain, and lower plasma testosterone (T) and 17β-estradiol (E2) levels. Olfactomedins and ependymin are candidates among the differentially expressed genes that may connect olfactory reception to the expression of *sgnrh* to regulate final maturation.

## Introduction

Reproductive homing migration, especially long distance semelparous migration, requires accurate interaction between the external and the internal environment, that is, between homing cues and the reproductive axis. Sensing the geographic position should be strongly linked to the stage of sexual maturation in both sexes to allow successful spawning of gametes. During the final stage of anadromous salmonid migration, olfactory cues guide the way to the spawning grounds [1]. Specific odorant factors of the natal river, that have been imprinted in particular areas of the nervous systems of juvenile salmon during downstream migration, are evoked by the adult salmon to recognize the natal river during the homing migration [2,3]. Indications exist that olfactory sensing of spawning ground cues simultaneously triggers the final maturation of the gonads [4,5,6]. In this study, we aimed at lifting the veil of the molecular basis of this process and the potential messengers to the reproductive axis, and at confirming the maturation status by assessing the changes that occur in the reproductive axis by tracing back signals from the maturing gonad and steroid messengers in the blood plasma, to the gonadotropins in the pituitary and to their regulation in the brain. This will be necessary to elucidate the molecular mechanisms and to investigate cause-effect relationships between the olfactory transcriptome and the sexual maturation stage in follow-up studies.

The reproductive axis in fish involves several levels of regulation of sexual maturation, at the levels of the brain, pituitary and gonad (BPG-axis). Development of the gonads in fish is regulated by pituitary gonadotropins (GTHs): Follicle Stimulating Hormone (FSH) and Luteinizing Hormone (LH). GTHs control gonad development directly and indirectly by regulating the production of gonadal steroids in both male and female fish [7]. Gonadotropin Releasing Hormone (GnRH) is a decapeptide neurohormone that stimulates the pituitary to produce and release GTHs and establishes a physical and functional connection between the brain and the pituitary through the innervation by GnRH neurons of many central nervous system (CNS) regions [8]. Salmon GnRH (sGnRH, or GnRH3 in zebrafish) is localized to the olfactory bulb-terminal nerve as well as to the preoptic area (POA) in the hypothalamus [9,10]. The sGnRH has been shown to elicit GTH-releasing activity and is considered to be the hypophysiotropic form [10,11].

**Fig 1:**
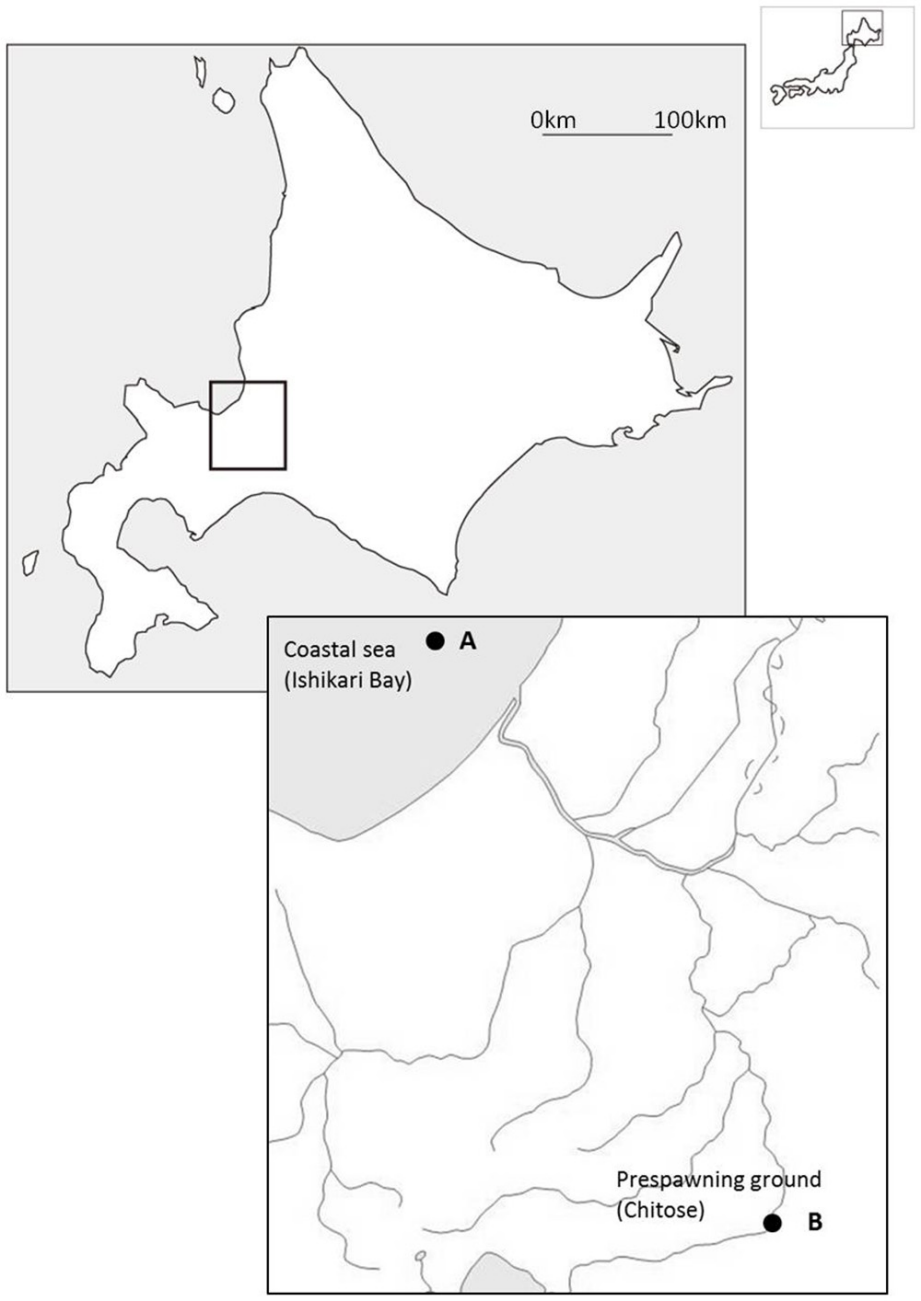
Sample sites of returning Chum salmon at the coastal sea of Ishikari Bay and 75 km upstream at the pre-spawning grounds of the Chitose River near Sapporo on Hokkaido, Japan.

During the final stages of the long term homing migration of salmonids, final maturation is likely triggered by one or more specific stimuli. Specifically, sGnRH present in the olfactory system, the terminal nerve and the POA is considered to play important roles in both salmon homing migration [12,13] and sexual maturation. This is also indicated by the effects of GnRH analog administration that include shortening of the homing migration and advancement of sexual maturation [14–18]. Hormone profiles have been studied [4–6] and one of the most important findings that supports a functional link between olfactory sensing and sexual maturation through sGnRH was that sGnRH levels in the telencephalon and serum testosterone (T) levels in male and female chum salmon showed a coincident peak at the branch point between the main river and the tributary that leads to the spawning grounds (Fig 1). These results support the idea that sGnRH, besides the hypothesized olfactory role, plays a role in GTH secretion in the pituitary of chum salmon. This strongly suggests a functional link between olfaction and reproductive regulation with sGnRH as direct effector. The need for fine-tuning between both processes points in the direction of sGnRH as a possible transducer of environmental olfactory signals to the GnRH POA neurons that regulate reproductive effects. These inputs may come from the olfactory rosettes where olfactory signals are received by olfactory receptors which are expressed in the sensory neurons. The efficient detection of dissolved odorants like amino acids, nucleotides, steroids, prostaglandins and bile acids (reviewed by Hino et al. [19]) plays a pivotal role in returning to the natal spawning grounds and is suggested to play a role in sexual maturation and reproductive success and, thus, directly contributes to Darwinian fitness. Still, many functional questions on olfactory reception in fish remain unanswered [19] including questions related to its link with reproductive neuroendocrinology.

This study assesses the olfactory transcriptome and the progression of sexual maturation in the brain-pituitary-gonadal axis of homing chum salmon. The expression of *sgnrh* and potential messenger genes as identified by RNAseq are investigated as important interactors between olfactory reception and final sexual maturation.

## Materials and Methods

### The choice of fish and the sample sites

This study was carried out in strict accordance with the recommendations in the Guide for the Care and Use of Laboratory Animals of the Japanese Governmental Law (No.105) and Notification (No.6). The protocol was approved by the Committee on the Ethics of Animal Experiments of Hokkaido University (Permit Number: 23–2).

Male and female chum salmon *Oncorhynchus keta* were caught on Oct. 2, 2011 by the Ishikari Bay Fisherman Association with a fixed fishing net at the coastal zone of the Pacific Ocean in the Ishikari Bay (43°23’ N, 141°13’ E; *N* = 10; 5 males and 5 females), and on Oct. 5, 2011 by the Hokkaido Salmon Propagation Association with traditional fish traps at a 75 km upstream site in Chitose River (pre-spawning site at 43°83’ N, 141°66’ E; Fig 1; also *N* = 10; 5 males and 5 females). All measurements were performed under clove oil anesthesia, and all efforts were made to minimize suffering before fish were sacrificed. The pre-spawning ground is located just 10 km downstream of the spawning ground. The rationale behind the choice of sampling sites was that in fish from the pre-spawning ground olfactory rosettes would be activated by the smell of the spawning ground, and that fish from Ishikari Bay could serve as a control group that was not yet activated by smell of the spawning ground.

### Biometry and tissue dissection

Fish were measured for body length and weight, sacrificed and sampled for blood. Fulton’s condition factor (K) was calculated according to the formula:

$$K=\;100\;\times \;\left(\text{BW}\;\times \;\text{B}{\text{L}}^{-3}\right),$$

with BW: body weight (g), BL: body length (cm).

Blood samples were taken from the caudal vein with heparin-flushed (10,000 IU ml^-1^) 10 ml syringes, which were immediately placed on ice. Blood was centrifuged for 15 min at 5,000 rpm and plasma was stored at -80°C for steroid measurements by time-resolved fluoroimmunoassay (TR-FIA). Fish were then dissected and sampled for olfactory rosettes, brain (sectioned in forebrain and post-brain areas) and pituitary. The forebrain sample included olfactory bulbs and telencephalon and the post-brain sample included the hypothalamus, midbrain tegmentum, medulla and cerebellum. Samples were flash frozen in liquid nitrogen and stored at -80°C for RNAseq and quantitative real time PCR (qPCR). Liver and gonad were sampled to determine their weights and to calculate the hepatosomatic index (HSI) and gonadosomatic index (GSI) according to the formulas:

$$\text{HSI}=\text{ (LW}\;\times \;{\text{(BW}-\text{LW)}}^{-1}\text{) }\times \;100\%,$$

with LW: liver weight (g);

$$\text{GSI}=\text{ (GW}\;\times \;{\left(\text{BW}-\text{GW}\right)}^{-1}\text{)}\;\times \;\text{100\%},$$

with GW: gonad weight (g), BW: body weight (g).

### Blood plasma steroids

For steroid measurements, blood plasma was extracted with diethyl ether and the extracts were dried at 50°C. After drying, the dry residues were reconstituted with a steroid assay buffer (50 mM Tris, 0.6% NaCl, 0.1% BSA, 20 μM DTPA, 0.01% Tween 40, pH 7.75). After extraction, plasma steroids (17α,20β-dihydroxy-4-pregnen-3-one (DHP), testosterone (T), 11-ketotestosterone (11-KT), 17β-estradiol (E2)) levels were measured by TR-FIA following the method of Yamada et al. [20]. In brief, steroids (0.05 μg/mL for DHP-BSA, T-BSA, 11-KT and 0.02 μg/mL for E2-BSA) were immobilized into 96-well microtiter plates (Wallac Oy, Turku, Finland). Fifty μL of the extracted sample and 150 μL of anti-steroid serum were dispensed into each well. Dilution factors of anti-steroid serum for DHP, T, 11-KT, E2 were 1:15,000, 1:7,000, 1:12,000 and 1:7,000, respectively. After reacting for 18 hours at 4°C, Eu-labelled IgG (DELFIA Eu-N1 labelled anti-rabbit IgG antibody (goat), PerkinElmer, Waltham, USA) was placed into each well and incubated for 1 hour at room temperature, and then thoroughly washed to remove any unbound Eu-labelled IgG. Fluorescence intensity from dissociated Eu was then measured by a microplate reader (Infinite F500, Tecan, Männedorf, Switzerland). The intra-assay coefficients of variation for DHP, T, 11-KT and E2 were 7.5%, 8.9%, 4.0% and 4.4%, respectively. The inter-assay coefficients of variation for DHP, T, 11-KT and E2 were 6.1%, 9.6%, 4.4% and 13.7%, respectively.

### Primers

Primers to the targeted genes were designed using the Genamics Expression software (www.genamics.com) and are given in Table 1. As housekeeping gene in all tissues (pituitary, post-brain, forebrain and rosettes), two different β-actin primers were used. Gonadotropin expression that was analysed in the pituitary included expression of the chum salmon glycoprotein hormone alpha-subunit (*gpα*), the FSHβ subunit (*fshβ*) and the LHβ subunit (*lhβ*). Primers to determine salmon-type GnRH (*sgnrh*) expression in the post-brain and forebrain were designed on basis of an *O*. *keta* genomic sequence that showed high similarity with *O*. *masou* mRNA for salmon-type GnRH precursor (Genbank D10946) and several other salmonid GnRH sequences.

**Table 1:** Nucleotide sequence of primers used for Q-PCR. Shown are the target genes, GenBank accession no., appropriate tissues for which expression was determined and primer sequences for the target genes.

Target gene	Genbank	Tissue		sequence (5´-3´)
*β-actin* (1)	n.a.*	pituitary, post-brain, forebrain	F	CAACTGGGAGGACATGGAGA
			R	GGTGCTCCTCTGGAGCCA
*β-actin* (2)	JX183093	post-brain, rosettes	F	GGCATCACACCTTCTACAACGAGC
			R	GGTCATCTTCTCCCTGTTGGCTT
*gpα*	M27652	pituitary	F	GCTTGGCAACAGATACTAGGCATT
			R	TGGGTCGCCTGTTCAAATGC
*fshβ*	M27153	pituitary	F	CATCATCGTGGAGAGAGAGGACTG
			R	AACGGGTATGAAGAAGGGCTCG
*lhβ*	M27154	pituitary	F	CAACTTTCTGCTGCCTGCTGA
			R	CCTAACATCCTGAAAGAGAGCCTG
*sgnrh*	DQ025624**	post-brain, forebrain	F	TAGCCAGCCATACGACCAGTG
			R	CAGGTGGTGGTGTTGGCGTTGGTA

* *β-actin* in-house designed primers were used;

** *sgnrh* primers were designed on basis of a *O*. *keta* genomic sequence that showed high similarity with *Oncorhynchus masou* mRNA for salmon-type gonadotropin-releasing hormone (sGnRH) precursor (Genbank D10946) and several other salmonid GnRHs.

### Real time PCR

RNA was isolated with TRIzol (Invitrogen, Carlsbad, USA) and concentrations were determined by spectrophotometry using a Genecuant (GE Healthcare, Uppsala, Sweden). DNAse treatment, reverse transcription and real time PCR were performed using protocols that are routinely used in Ueda’s laboratory at the Hokkaido University.

RNA samples containing 5 μg of RNA were DNAse treated with RQ1 DNAse (Promega, Madison, USA) and reverse transcribed using PrimeScript^tm^ II RTase (Takara Bio, Otsu, Japan) in a Takara PCR thermal cycler (Takara Bio, Otsu, Japan), according to the manufacturers’ protocols. cDNA samples were diluted 1:25 and used as a template. The reactions (20 μl final volume) contained 10 μl of SYBR GreenER qPCR SuperMix (Invitrogen), 500 nM concentration of forward and reverse primers and 5 μl of cDNA. Reactions were run in a Mx3000P Real-Time PCR Detection System (Stratagene) using the following protocol: 2 min at 50°C, 8 min at 95°C, followed by 40 cycles of 15 sec denaturation at 95°C and 30 sec at the corresponding melting temperatures, and a final melting curve of 81 cycles from 55°C to 95°C (0.5°C increments every 10 sec). Samples (*N* = 8 per site: four males and four females) were run in triplicate and fluorescence was measured at the end of every extension step. Fluorescence readings were used to estimate the values for the threshold cycles (Ct). The Ct values were normalized for each gene against those obtained for the housekeeping gene *β-actin* on the same plate. Normalized Ct values were expressed as fold changes (fc) using the relative quantification method [21], calculated for fish from the pre-spawning ground relative to those from Ishikari Bay.

### RNA sequencing

Remaining RNA samples of olfactory rosettes in RNAse-free dH_2_O were transferred to Leiden (The Netherlands) for RNAseq by adding 1:10 volume of 3M sodium acetate at a pH of 4.8 and 2.5 volumes of 100% ethanol to each of the samples. For RNAseq, 12 chum salmon olfactory rosette RNA samples were used: three females and three males from each of the sites. In Leiden, RNA was precipitated by centrifugation, washed in 70% ethanol and dissolved again in RNAse free dH2O. RNA concentrations as determined with a Bioanalyzer were between 28 and 115 ng μl^-1^ and RIN values between 5.8 and 9.5. Illumina multiplexed RNAseq libraries were prepared from 2 μg total RNA using the Illumina TruSeq^TM^ RNA Sample Prep Kit v2 according to the manufacturer’s instructions (Illumina Inc.). Library concentrations were between 7.7 and 42.5 ng μl^-1^ corresponding to molarities between 39.5 and 214.5 nmol l^-1^. All RNAseq libraries were sequenced on an Illumina HiSeq2500 sequencer as 2 x 50 nucleotides paired-end (PE50) reads according to the manufacturer’s protocol. Image analysis and base calling were done by the Illumina pipeline. The number of PE50 reads varied from 11.2 to 37.9 million reads per sample, with an average of 20.8 million reads per sample (Table A in S1 File). The raw RNAseq data of the twelve olfactory rosette samples have been deposited in the NCBI GEO repository with accession number GSE72390.

Since there was no reference genome of chum salmon (*O*. *keta*) at the time of data analysis, two strategies were used for quantitative analysis of the olfactory placode RNAseq data sets:

In strategy 1, RNAseq reads from the 12 individual samples were aligned to 48,223 *Salmo salar* cDNA sequences (downloaded from NCBI) using TopHat (version 2.0.5) [22]. A preliminary test showed that *O*. *keta* and Atlantic salmon (*Salmo salar*) cDNA sequences are approximately 95% identical. Therefore, the reference alignment was done allowing 3 mismatches per 50 nt read (3 mismatches ~ 94% identity). The resulting files were filtered using SAMtools (version 0.1.18) [23] to exclude secondary alignment of reads. Via this strategy about 15% of the chum salmon RNAseq reads could be mapped against the *S*. *salar* reference database (Table A in S1 File). For statistical comparison of gene expression levels between groups, aligned fragments per predicted gene were counted from SAM alignment files using the Python package HTSeq (version 0.5.3p9) [24]. In order to make comparisons across samples possible, these fragment counts were corrected for the total amount of sequencing performed for each sample. As a correction scaling factor, we employed library size estimates determined using the R/Bioconductor (release 2.11) package DESeq [25]. Read counts were normalized by dividing the raw counts obtained from HTSeq by its scale factor.

In strategy 2, CLC bio’s *de novo* assembler was used to generate cDNA contigs from a total of ~250 million PE50 reads derived from all 12 RNAseq libraries. This resulted in 98,542 cDNA contigs corresponding to mRNAs that were expressed in the *O*. *keta* olfactory placodes and ranging in size from 200 to 17,327 nt (Table B in S1 File). The *de novo* cDNA contigs were annotated to known genes via custom BLAST searches against four reference databases: (a) a UNIPROT protein database (34.7% hits), (b) a Teleost Refseq mRNA database (26.3% hits), (c) a Teleost "all mRNA" database (72.2% hits) and (d) the *Danio rerio* Zv9 genome (7.1% hits). RNAseq reads from the 12 individual samples were then aligned to this *de novo* assembled chum salmon olfactory rosetta cDNA reference database as described above under "strategy 1". About 60% of the RNAseq reads could be mapped against the *de novo* assembled reference database (Table A in S1 File). Statistical comparison between groups of fish from the prespawning ground vs. fish from the coastal sea site was done using HTseq and DESeq as described above under "strategy 1". Then, for both strategies, olfactory genes were identified by searching for the term 'olfact'. Olfactory genes resulting from strategy 2 were then compared with salmonid and *O*. *keta* NCBI sequences with the megablast option.

### Statistical analysis

Normal distribution was checked by Kolmogorov Smirnoff tests. Pair-wise group comparisons, either comparing all fish from the pre-spawning grounds vs. those from Ishikari Bay or comparing sex-specifically, were performed to check: 1) potential differences in size and housekeeping gene *β-actin* expression, and 2) potential differences in the experimental parameter set consisting of K, HSI and GSI; plasma steroids T, 11-KT, E2 and DHP; expression of pituitary *gpα*, *fshβ* and *lhβ*; expression of forebrain and post-brain *sgnrh*. As for the relevant comparisons, no significant differences existed in BW between the groups from both sites, between females from both groups and between males from both groups. No differences in housekeeping gene *β-actin* expression for the different tissues existed between groups. Potential differences in experimental parameters were analysed by pair-wise testing with a Student’s t-test or Mann-Whitney U-test and were considered significant when P<0.05. Bivariate Pearson correlations among all measured parameters were tested one-tailed in the direction of advance of maturation on log transformed data. All statistical analyses were performed with SPSS 19.0. Values are expressed as average ± standard error (SE).

## Results

### Condition factor, HSI and GSI

Chum salmon that were caught in the coastal sea at Ishikari Bay had a body-weight (BW) of 2,842 ± 217 g (mean ± SE) and were not significantly different in size from those caught at the pre-spawning ground in Chitose River that weighted 2,940 ± 186 g. Body-length (BL) of fish caught at the coastal sea was 71 ± 1 cm and slightly longer than those caught at the pre-spawning ground at 66 ± 1cm. No significant differences existed in BW between the groups from both sites, between females from both sites and between males from both sites (Table 2). Females caught at the pre-spawning ground were shorter than those caught in the coastal sea which caused the overall difference in length between both groups. The males caught at the prespawning ground were significantly larger and heavier than the females from this site.

**Table 2:** Size (fork length FL and body weight BW), condition factor (K), hepatosomatic index (HSI) and gonadosomatic index (GSI) of male and female chum salmon at the coastal sea (Ishikari Bay) and at the pre-spawning ground (Chitose). Significant differences between fish from both sites are indicated with * for P<0.05 and ** for P<0.01.

		coastal sea	prespawning ground
males	N	5	5
	FL(cm)	70 ± 1	68 ± 1
	BW(g)	2701 ± 306	3335 ± 139
	K	0.784 ± 0.083	1.066 ± 0.035**
	HSI	1.52 ± 0.17	2.36 ± 0.13**
	GSI	5.88 ± 0.60	4.25 ± 0.40*
females	N	5	5
	FL(cm)	72 ± 1	63 ± 2**
	BW(g)	2983 ± 329	2544 ± 241
	K	0.783 ± 0.058	1.004 ± 0.051*
	HSI	2.55 ± 0.14	1.78 ± 0.37*
	GSI	17.26 ± 2.75	20.27 ± 2.43

Both for females and males, the condition factor (K) was significantly higher for fish caught at the pre-spawning ground than those caught in the coastal sea (Table 2). For females and males in the coastal sea, K values were similar (0.78 ± 0.06 and 0.78 ± 0.08, respectively), as with the higher values at the pre-spawning ground (1.00 ± 0.05 and 1.07 ± 0.03, respectively). HSI was significantly lower for females and higher for males from the pre-spawning ground vs. those from the coastal sea site (Table 2). GSI values showed an opposite change as compared to HSI with higher values in females (20.27 ± 2.43 vs. 17.26 ± 2.75; ns) and significantly lower values in males from the pre-spawning ground vs. the coastal sea (4.25 ± 0.40 vs. 5.88 ± 0.60, respectively; Table 2).

### Plasma steroid levels

**Fig 2:**
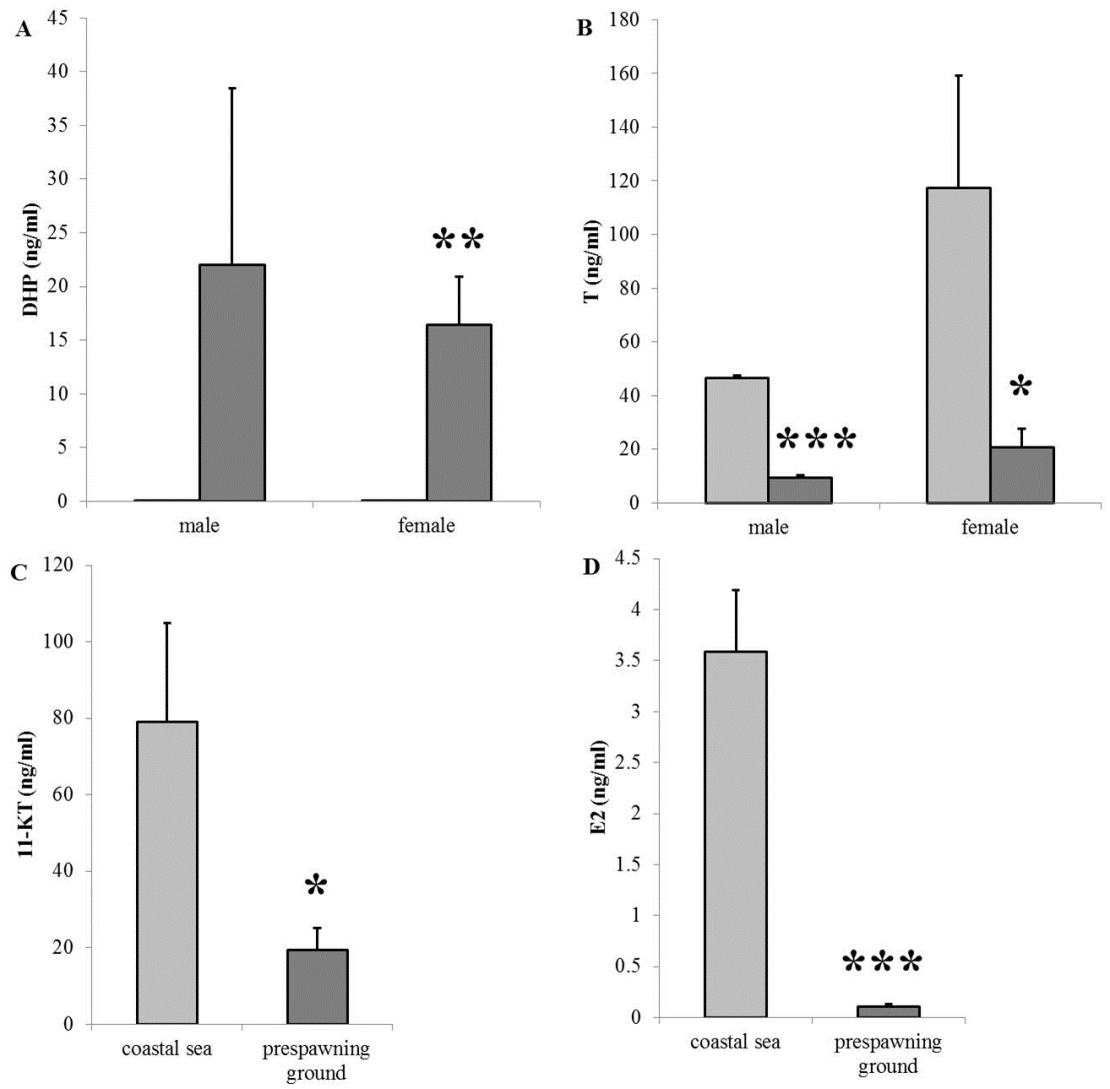
Plasma steroid levels of male and female chum salmon at the coastal sea (Ishikari Bay) and at the pre-spawning ground (Chitose). (A) 17α,20β-dihydroxy-4-pregnen-3-one (DHP) in males and females at the coastal sea (light grey) and the pre-spawning ground (dark grey). DHP levels are much higher at the pre-spawning ground than at the coastal sea, significant only for the females. (B) Testosterone (T) in males and females at the coastal sea (light grey) and the pre-spawning ground (dark grey). Both in males and females, T levels are significantly lower at the pre-spawning ground vs. the coastal sea. (C) 11-ketotestosterone (11-KT) levels in males are significantly lower at the pre-spawning ground vs. the coastal sea. (D) 17β-estradiol (E2) levels in females are significantly lower at the pre-spawning ground vs. the coastal sea.

In males, plasma DHP levels increased from 0.064 ± 0.034 ng ml^-1^ at the coastal sea to 22 ± 16 ng ml^-1^ at the pre-spawning grounds (Fig 2A), representing a 344-fold increase although not significant due to high individual variation. In females, plasma DHP levels increased significantly from 0.022 ± 0.068 ng ml^-1^ to 16 ± 4 ng ml^-1^, representing a 747-fold increase. In contrast, T levels dropped significantly in males and females when comparing levels between fish from the coastal sea and the pre-spawning ground (Fig 2B). T levels decreased approximately 5-fold from 46 ± 1 ng ml^-1^ to 9 ± 1 ng ml^-1^ in males, and from 117 ± 42 ng ml^-1^ to 20 ± 7 ng ml^-1^ in females.

In males, levels of 11-KT decreased significantly in fish from the coastal sea to pre-spawning ground sites (from 79 ± 26 ng ml^-1^ to 19 ± 6 ng ml^-1^) (Fig 2C), in line with the T levels. Also, E2 levels in females decreased significantly from the coastal sea to pre-spawning ground, from 3.6 ± 0.6 ng ml^-1^ to 0.11 ± 0.02 ng ml^-1^, respectively (Fig 2D).

### Gonadotropin expression in the pituitary

**Fig 3:**
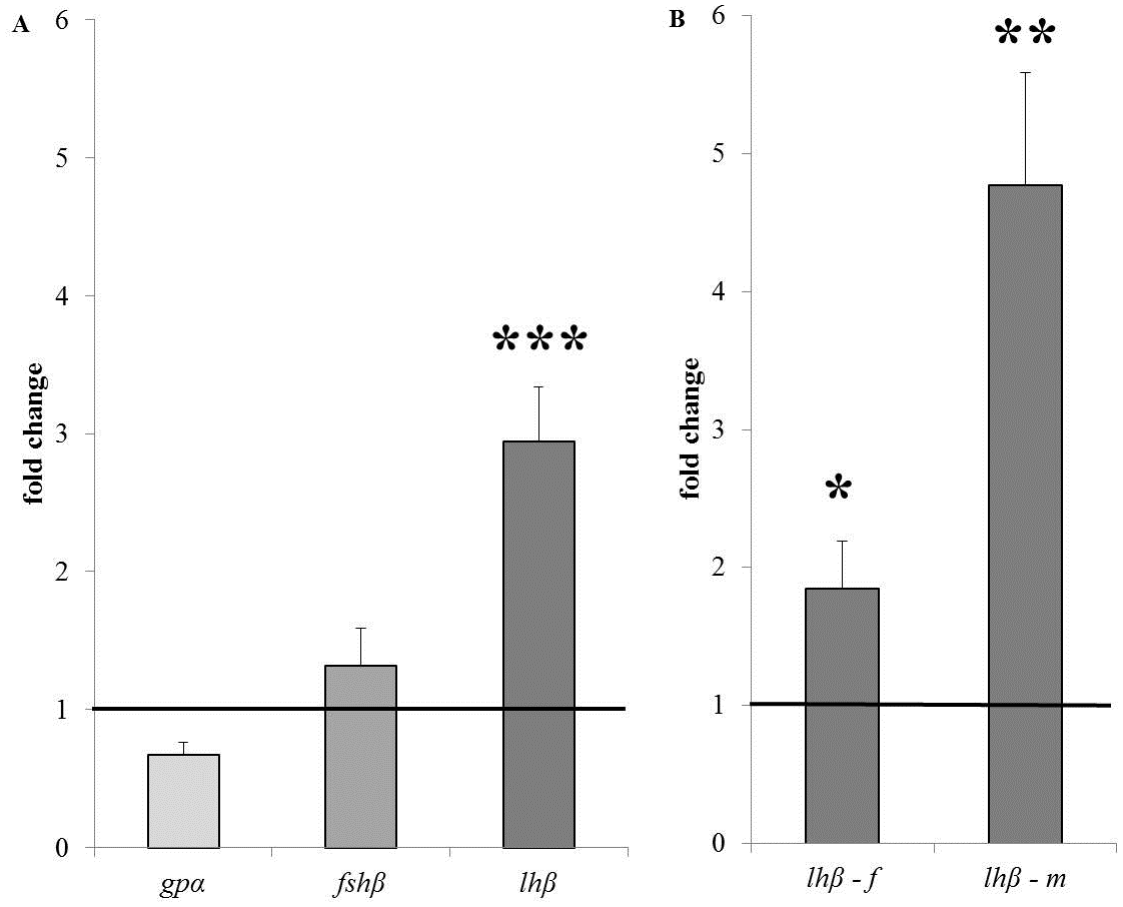
*Glycoprotein α* (*gpα*), *fshβ* and *lhβ* expression in the pituitary of chum salmon. (A) Expression of each of these target genes (normalized vs. the expression of the housekeeping gene) is shown as fold change of fish at the pre-spawning ground vs. fish at the coastal sea (set at fc 1 as indicated by the line). *lhβ* expression is significantly higher at fc 2.94 ± 0.40. (B) *lhβ* expression is significantly higher in females (f) at fc 1.85 ± 0.34 and males (m) at fc 4.77 ± 0.82.

Expression of the common subunit *gpα* and of *fshβ* in the pituitary, as normalised vs. the expression of housekeeping gene *β-actin*, was not significantly different between fish from the pre-spawning and the coastal sea site (Fig 3A): fc -1.49 ± 0.20 and 1.32 ± 0.27, respectively. Expression of *lhβ* was, however, much higher in fish from the pre-spawning ground at fc 2.94 ± 0.40. Up-regulated expression of *lhβ* was significant in both females at fc 1.85 ± 0.34 and males at fc 4.77 ± 0.82 (Fig 3B).

### *Salmon gnrh* in the brain

**Fig 4:**
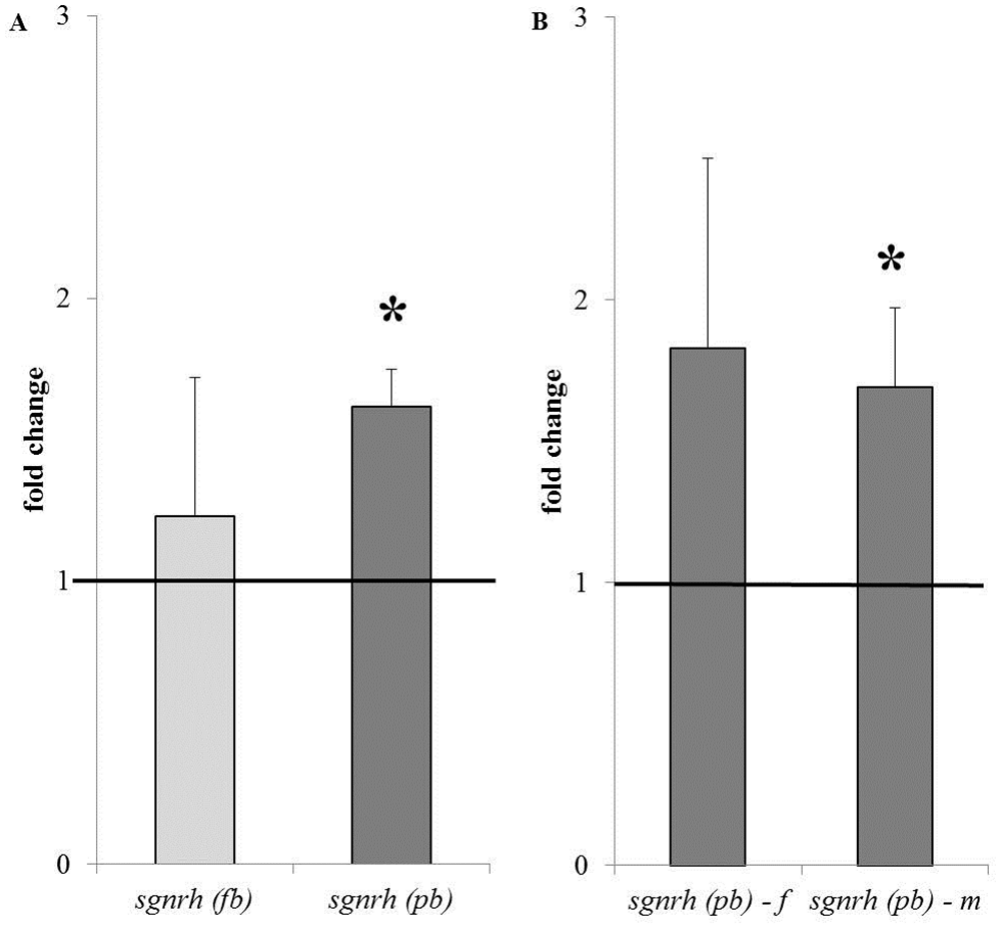
*Salmon gonadotropin releasing hormone* (*sgnrh*) expression in the brain of chum salmon. (A) Expression of *sgnrh* (normalized vs. the expression of the housekeeping gene) is shown as fold change of fish at the pre-spawning ground vs. fish at the coastal sea (set at fc 1 as indicated by the line). *sgnrh* expression is significantly higher at fc 1.62 ± 0.26. (B) *sgnrh* expression is higher in both sexes but significantly higher in males. pb = post-brain; fb = forebrain, f = female; m = male.

Expression of *sgnrh* in the forebrain was not different between fish from the pre-spawning ground vs. fish from the coastal sea but *sgnrh* expression in the post-brain was significantly higher in fish from the pre-spawning ground at fc 1.69 ± 0.28 (Fig 4A). Expression of *sgnrh* in the post-brain appeared to be higher in females but was only significantly higher in males (Fig 4B).

### Bivariate correlation analysis

Bivariate Pearson correlations among all measured parameters are given in Table C in S1 File. Among the significant bivariate correlations were all maturation parameters. A positive correlation existed between *sgnrh* in the post brain and *lhβ* in the pituitary.

### RNAseq of the olfactory rosettes

Strategy 1, mapping reads against 48,223 *S*. *salar* NCBI sequences, showed that 29,665 *S*. *salar* sequences (allowing three mismatches) were expressed: expression of 15,286 genes was up-regulated and expression of 14,379 genes was down-regulated. Of those expressed sequences, 3,234 genes were significantly differentially expressed (p<0.05; 11%): expression of 1,792 genes was up-regulated and expression of 1,442 genes was down-regulated. Table D in S1 File shows the 226 most relevant genes: expression of 131 genes was up-regulated and expression of 95 genes was down-regulated. Among the differentially expressed genes was *ependymin* (gi|221219751), the expression of which was up-regulated at fc 3 in fish from the pre-spawning ground as compared to those from the coastal sea site.

When specifically searching for olfactory genes, 69 of the 96 *S*. *salar* NCBI olfactory genes were found to be expressed in chum salmon olfactory rosettes. Expression of 36 of these genes was up-regulated and 7 of these genes were significantly differentially expressed in a tight range between fc 1.53 and 2.06: *MOR500-1* at fc 2.06; *MOR120-2* at fc 1.74; *MOR120-1* at fc 1.74; *MOR500-3* at fc 1.73; *MOR112-1* at fc 1.73; *MOR500-2* at fc 1.63 and *MOR115-4* at fc 1.53 (P<0.05; Table E in S1 File). Expression of 33 olfactory genes was down-regulated and 1 of these genes was significantly differentially expressed: *olfactory receptor family C subfamily 13 member 1 gene* at fc -1.37.

Strategy 2, comparing de novo *O*. *keta* contigs with four reference databases, revealed:

1. BLASTX hits found for 34,177 of the 98,542 *O*. *keta* contigs against UNIPROT database (34.7%);
2. BLASTN hits for 25,941 of the 98,542 *O*. *keta* contigs against Teleost Refseq (137,148 sequences)(26.3%);
3. BLASTN hits for 71,183 of the 98,542 *O*. *keta* contigs against all Teleost RNAs (1,475,267 sequences)(72.2%);
4. BLASTN hits for 6,976 of the 98,542 *O*. *keta* contigs against the *Danio rerio* genome (7.1%).

An 'olfact' search among these hits resulted in 43 genes (Table F in S1 File). Three of these genes showed high identity with salmonid and *O*. *keta* NCBI sequences. Seventeen of these genes showed high identity with salmonid NCBI sequences but comparing with *O*. *keta* NCBI sequences gave no results. Finally, twenty-seven of the ‘olfact’ genes gave no results for neither salmonid NCBI sequences nor *O*. *keta* NCBI sequences.

Of these 43 genes, nine were differentially expressed of which four salmonid genes were the same as those found in strategy 1, three salmonid genes were not found in strategy 1 and two gave no result in the salmonid megablast (Table F in S1 File). The latter five genes, with the eight genes found by strategy 1, bring the total number of differentially expressed olfactory genes to thirteen (Table 3). Of these five genes, expression of four genes was up-regulated in fish from the pre-spawning ground vs. those from the coastal sea site: *OMP MOUSE*/ *Oncorhynchus nerka sOMP2* at fc 2.46; *O52K1 HUMAN/ Salmo salar odorant receptor ASOR1-like* at fc 1.73 and two olfactomedins (see below), and expression of one gene was down-regulated: *O51F2 HUMAN* at fc -1.79, a novel salmonid gene.

**Table 3:** Differentially expressed olfactory genes. Shown are the genes for which the expression was up-regulated (green; N = 11) and down-regulated (red; N = 2); the NCBI gene identifier gi; the gene description; the P-value and the fold change (fc).

GI version	Description	pval	fc
gi|238624075	Oncorhynchus nerka sOMP2 mRNA for salmon olfactory marker protein 2, complete cds	0.000	2.46
gi|378747413	Salmo salar main olfactory receptor family E subfamily 500 member 1 (MOR500-1) gene, partial cds	0.003	2.06
gi|562022	OLF3A DANRE Olfactomedin-like protein 3A	0.017	1.86
gi|224587432	Salmo salar clone ssal-rgf-519-289 Olfactomedin-4 precursor putative mRNA, pseudogene cds	0.027	1.75
gi|378747427	Salmo salar main olfactory receptor family E subfamily 120 member 2 (MOR120-2) gene, partial cds	0.035	1.74
gi|378747425	Salmo salar main olfactory receptor family E subfamily 120 member 1 (MOR120-1) gene, partial cds	0.016	1.74
gi|378747417	Salmo salar main olfactory receptor family E subfamily 500 member 3 (MOR500-3) gene, partial cds	0.018	1.73
gi|378747437	Salmo salar main olfactory receptor family A subfamily 112 member 1 (MOR112-1) gene, partial cds	0.019	1.73
gi|185133823	Salmo salar odorant receptor ASOR1-like (LOC100136423), mRNA	0.035	1.73
gi|378747415	Salmo salar main olfactory receptor family E subfamily 500 member 2 (MOR500-2) gene, partial cds	0.020	1.63
gi|378747445	Salmo salar main olfactory receptor family F subfamily 115 member 4 (MOR115-4) gene, complete cds	0.032	1.53
gi|329130711	Salmo salar olfactory receptor family C subfamily 13 member 1 gene, complete cds	0.045	0.73
gi|119694	O51F2 HUMAN Olfactory receptor 51F2	0.004	0.56

Among the genes that resulted from an 'olfact' search were a large number of olfactomedins (1, 2A, 2B, 3, 3A, 4) that are extracellular matrix proteins in the olfactory neuroepithelium [26]. Two of the olfactomedins were differentially expressed between fish from the pre-spawning ground vs. those from the coastal sea site: *Olfactomedin-4* (*olfm4*; mouse) at fc 1.75 and *Olfactomedin-like protein 3A* (*olf3A*; zebrafish) at fc 1.86 (Table 3). Megablasting *olfm4* against salmonid sequences revealed high similarity with the *Salmo salar clone ssal-rgf-519-289 Olfactomedin-4 precursor putative mRNA*, *pseudogene cds* (97%) but did not provide a hit for chum salmon. Megablasting *olf3A* against salmonid sequences did not give any result suggesting that this gene is new for salmonids.

## Discussion

In order to gain insight into the expression of the full array of olfactory genes and to identify novel olfactory genes of importance which is crucial for the advancement of homing studies, this study reports on RNAseq of chum salmon olfactory rosettes. The olfactory transcriptomes were compared between male and female fish from the pre-spawning grounds that are hypothesized to have recognized specific odorant factors of the natal rivers (‘activated’) vs. ‘non-activated’ fish from the coastal sea. This comparison has revealed novel genes and their potential importance in homing-related olfaction. Changes along the brain-pituitary-gonadal axis have been assessed to confirm that fish at the spawning grounds had much advanced in their sexual maturation state in comparison with fish in the ocean and that they had reached final maturation. Assessing these changes was performed applying a diversity of mostly established techniques and available information in order to confirm the advanced maturation of fish near the spawning grounds vs. fish from the coastal sea. The paired insights that have been gained in the processes of olfaction and sexual maturation in homing chum salmon allow us to further consider a link between both processes.

### The plasma steroids: final maturation at the pre-spawning ground

Lower HSI values and plasma E2 and T levels in female fish from the pre-spawning ground vs. those from the coastal sea indicate the completion of vitellogenesis (Table 2, Fig 2). DHP levels in males and females from the pre-spawning ground were much higher than in fish from the coastal sea. Elevated plasma DHP levels are a strong indicator of final maturation, suggesting that both males and females at the pre-spawning ground were more advanced reproductively than coastal sea fish and had initiated the final maturation process (Fig 2A). This is also reflected by an increased condition factor.

### The pituitary: regulation of final maturation and feedback by plasma steroids

The higher *lhβ* expression in the pituitaries of fish from the pre-spawning ground vs. those from the coastal sea (Fig 3) fits well with the progression of gametogenesis towards final maturation. LH is required to initiate the processes of final maturation and ovulation in salmonid fish [27,28]. Furthermore, as expected, the higher *lhβ* expression was not accompanied by higher expression of *gpα* nor any changes in *fshβ* expression.

The higher *lhβ* expression in the pituitaries in fish from the pre-spawning ground vs. those from the coastal sea may result from the lower plasma androgen and E2 levels, implying less negative feedback on the pituitary in the production of LH. Both females and males showed decreased plasma steroid levels and increased *lhβ* expression when comparing fish from the pre-spawning ground vs. those from the coastal sea but the higher *lhβ* expression was most apparent in males (Fig 3), probably indirectly resulting from the decrease in GSI.

### The brain: regulation of GTHs by sGnRH

In salmonids, GTH production and release is stimulated by sGnRH. Primers were designed against an *O*. *keta* genomic sequence that showed high similarity with *O*. *masou* mRNA for sGnRH precursor and several other salmonid GnRH sequences. We can confirm that this *O*. *keta* genomic sequence is chum salmon sGnRH because it is identical to the sGnRH sequence for chum salmon that was published by Kim et al. [29](Genbank JX183101). sGnRH is localized to the olfactory bulb-terminal nerve as well as to the POA [12] and, due to the way the dissection of the brain was performed in this study, *sgnrh* expression can be expected in both the forebrain (including the olfactory bulb and the telencephalon) and the post brain (including the POA) samples. In earlier studies by Yamada et al. [30] and by Makino et al. [6], sGnRH levels first showed a peak in the olfactory bulb from the coastal sea to the mouth of Ishikari River and then in the telencephalon at the branch point between the Ishikari River and the Chitose River that leads to the spawning grounds (reviewed by Hayashida et al. [31]). In this study, chum *sgnrh* expression was detected in forebrain and post brain of fish that had already migrated further upstream the Chitose River to the pre-spawning ground (Fig 1). In these fish we found that the expression of *sgnrh* was significantly up-regulated in the post brain (Fig 4), thus in the POA, which would support sGnRH signalling from forebrain to post brain, from the olfactory bulb to telencephalon and, subsequently, to the POA. As the functional relation would imply, chum *sgnrh* was likely positively correlated with LH production in this study, specifically supported by the positive correlation between *sgnrh* expression in the post brain and *lhβ* expression in the pituitary (Table C in S1 File).

### The olfactory transcriptome: receiving and transmitting olfactory signals from the spawning grounds

Our study is aimed at investigating the olfactory transcriptome in relation to homing migration in fish, as recently performed for the Japanese Grenadier Anchovy (*Coilia nasus*; [32]) and European eel (*Anguilla anguilla*; [33]), but ours is the first study aimed at salmonid homing. Deep RNA sequencing revealed the expression of 75 known and 27 unknown salmonid genes that have relation to olfaction. Three super-families of fish olfactory receptors exist: the vomeronasal receptor-like genes V2R-like expressed by *olfc* genes and V1R-like expressed by *ora* genes, and the main olfactory receptors expressed by *mOR* genes [34–36]. In the present study, all three superfamilies were represented among the expressed genes. Thirteen olfactory genes were differentially expressed between chum salmon from the pre-spawning ground vs. those from the coastal sea site (Table 3) suggesting an important role of these genes in homing. The significantly up-regulated olfactory genes were all *mOR* genes (strategy 1: *Salmo salar* NCBI sequences) expressing the main olfactory receptors, an olfactory marker protein and an odorant receptor. The significantly down-regulated genes were a V2R-like expressed by *olfc* and a novel salmonid olfactory receptor. In addition to the receptors, three potential signalling molecules were expressed in the olfactory rosettes: *ependymin*, *olfactomedins* and *sgnrh*. Ependymin is a myelin-associated and hormonally-induced glycoprotein involved in learning and consolidation of long-term memory [37–40]. In this study, ependymin expression was up-regulated at fc 3 in the homing salmons suggesting an important role in the olfactory memory. Thereby ependymin is hypothesized to be involved in the retrieval of olfactory imprinting memory, once retained as juvenile migrating downstream from their natal stream to the ocean. Also in fall- and spring-run Chinook salmon, ependymin exhibited much higher expression levels than in an individual from the ocean [41]. Recently, ependymin (in this case the ependymin II precursor) was shown to play an important role as neural mediator in the olfactory processing of sex pheromones in the telencephalon of goldfish [42]. Ependymin and sGnRH expression were increased after PGF2α exposure implying a similar functional relationship between olfactory reception and sexual maturation as in homing salmon. Although ependymin may certainly signal from olfactory sensing of the spawning ground to the reproductive axis, Churcher et al. [33] reported on an increase in the levels of mammalian ependymin-related protein (MERP) in the brains of experimentally matured male eels showing that changes could be secondary to hormonal changes during sexual maturation.

Most *olfactomedins* were highly expressed in the Chum salmon olfactory rosettes. Two *olfactomedins*, *olfm4* and *olf3A* were differentially expressed, suggesting a potentially important role in sensing the spawning grounds during homing. Interestingly, like *ependymin*, most *olfactomedins* are expressed in neural tissues and they may serve as receptors and as modulators of signalling pathways [43]. As such they are certainly candidates to play an important role in conveying olfactory signals to other parts of the brain, e.g. in the pathway that functionally connects olfaction to sexual maturation through sGnRH. *Olfactomedin 4* (*S*. *salar* NCBI gi|224587432) and the novel salmonid gene *olf3A* are therefore worthwhile to investigate more thoroughly in mature homing fish, in particular in relation to the expression of sGnRH at the mRNA and protein levels

Finally, RNAseq also showed that *sgnrh* was hardly expressed in the olfactory rosettes implying that the olfactory cues may not be immediately linked to maturation through sGnRH but that intermediate messengers like ependymin and olfactomedins may play a role.

### Conclusions and perspectives

From this study it can be concluded that at the pre-spawning ground, the olfactory reception of spawning ground specific cues in the olfactory rosettes appeared to be activated as evidenced by the up-regulated expression of eleven olfactory genes. A clear progression towards final maturation occurred in homing chum salmon from the coastal sea to 75 km upstream the rivers at the pre-spawning ground, mainly characterised by higher plasma DHP levels, higher pituitary *lhβ* expression and *sgnrh* expression in the post brain, and lower plasma T and E2 levels. Olfactomedins and ependymin may connect olfactory reception to the expression of *sgnrh* in the post brain to regulate final maturation at the pre-spawning ground.

Now that these olfactory genes have been identified, their role can be thoroughly examined in larger sample sizes and with a broader geographical coverage. Also, the geographical pattern of olfactory gene expression along the migration route may then be revealed. Gained insights will invite scientists to perform molecular and behavioural studies on the functional role of specific olfactory genes. Male and female olfactory gene expression fingerprints may reveal sex-specific differences in pheromone sensing and sensing of spawning ground specific cues.

## Supporting Information

S1 File **Table A- Reads.**; **Table B- Results of de novo assembly of 12 x PE50 RNAseq datasets.**; **Table C- Bivariate Pearson correlations.** Given are the measured parameters and the Pearson correlation, the significance and the number (N) of individuals for which values were available to perform the analysis for each of the measured parameters. Significance is also indicated by * = P<0.05 and ** = P<0.01. Pearson correlation was analysed one-tailed (in the direction of advance of maturation) of log transformed data. Abbreviations: bl = body-length; bw = body-weight; k = condition factor; gsi = gonadosomatic index; hsi = hepatosomatic index; sgnrhfb = *salmon-type gonadotropin-releasing hormone* in the forebrain; sgnrhpb = *sgnrh* in the post brain; gp = *glycoprotein hormone alpha-subunit*; fsh = *fshβ subunit*; lh = *lhβ subunit*; e2 = 17β-estradiol; t = testosterone; kt = 11-ketotestosterone; dhp = 17α,20β-dihydroxy-4-pregnen-3-one.; **Table D- The 226 most relevant differentially expressed genes.** Considered as relevant was expression of contigs as based on very stringent criteria: without sasaskin mRNA sequences; expressed in fish of both sites; with p<0.01 and with 2<fc<-2. The expression of 131 genes was up-regulated (green) and expression of 95 genes was down-regulated (red). Shown are the NCBI gene identifier gi; accession number; gene description; P-value and fold change (fc).; **Table E- The 69 expressed olfactory genes resulting from strategy 1 (mapping reads against 48,223 S. salar NCBI sequences allowing three mismatches).** Expression of 36 of these genes was up-regulated (green) of which for 7 genes significantly (bold). Expression of 33 of the genes was down-regulated (red) of which for 1 gene significantly (bold). Shown are the NCBI gene identifier gi; accession number; gene description; P-value and fold change (fc).; **Table F- The 43 expressed olfactory genes resulting from strategy 2 (comparing de novo contigs with four reference databases UNIPROT, Teleost Refseq, all Teleost RNAs, *Danio rerio* genome).** Shown are the gene id; the function description; the P-value; the fold change (fc); the result of the salmonid megablast as NCBI gene identifier gi; gene description and the id level. Differentially expressed genes are given in bold.
(DOCX)
