# Supplementary material for: The Olfactory Transcriptome and Progression of Sexual Maturation in Homing Chum Salmon Oncorhynchus keta
Source: PLoS One. 2015 Sep 23;10(9):e0137404. doi: 10.1371/journal.pone.0137404 (PMC4580453; doi:10.1371/journal.pone.0137404)
Supplement: S1 File — Table A- Reads.; Table B- Results of de novo assembly of 12 x PE50 RNAseq datasets.; Table C- Bivariate Pearson correlations. Given are the measured parameters and the Pearson correlation, the significance and the number (N) of individuals for which values were available to perform the analysis for each of the measured parameters. Significance is also indicated by * = P<0.05 and ** = P<0.01. Pearson correlation was analysed one-tailed (in the direction of advance of maturation) of log transformed data. Abbreviations: bl = body-length; bw = body-weight; k = condition factor; gsi = gonadosomatic index; hsi = hepatosomatic index; sgnrhfb = salmon-type gonadotropin-releasing hormone in the forebrain; sgnrhpb = sgnrh in the post brain; gp = glycoprotein hormone alpha-subunit; fsh = fshβ subunit; lh = lhβ subunit; e2 = 17β-estradiol; t = testosterone; kt = 11-ketotestosterone; dhp = 17α,20β-dihydroxy-4-pregnen-3-one.; Table D- The 226 most relevant differentially expressed genes. Considered as relevant was expression of contigs as based on very stringent criteria: without sasaskin mRNA sequences; expressed in fish of both sites; with p<0.01 and with 2<fc<-2. The expression of 131 genes was up-regulated (green) and expression of 95 genes was down-regulated (red). Shown are the NCBI gene identifier gi; accession number; gene description; P-value and fold change (fc).; Table E- The 69 expressed olfactory genes resulting from strategy 1 (mapping reads against 48,223 S. salar NCBI sequences allowing three mismatches). Expression of 36 of these genes was up-regulated (green) of which for 7 genes significantly (bold). Expression of 33 of the genes was down-regulated (red) of which for 1 gene significantly (bold). Shown are the NCBI gene identifier gi; accession number; gene description; P-value and fold change (fc).; Table F- The 43 expressed olfactory genes resulting from strategy 2 (comparing de novo contigs with four reference databases UNIPROT, Teleost Refseq, all Teleost RN [file pone.0137404.s001.docx]

**Table A: Reads.**

| Sample code | # PE50 reads | # PE50 reads aligned to *S. salar* reference (max 3 mismatches) | # PE50 reads aligned to *O. keta* de novo reference |
| --- | --- | --- | --- |
| Ishikari Bay, female 1, OE I4 | 37,860,760 | 5,579,526 (14.7 %) | 22,958,610 (60.6 %) |
| Ishikari Bay, female 2, OE I9 | 30,113,272 | 4,618,835 (15.3 %) | 18,432,842 (61.2 %) |
| Ishikari Bay, female 3, OE I10 | 20,434,586 | 3,171,405 (15.5 %) | 12,595,495 (61.6 %) |
| Ishikari Bay, male 1, OE I2 | 11,181,296 | 1,688,255 (15.1 %) | 7,038,484 (63.0 %) |
| Ishikari Bay, male 2, OE I5 | 17,152,900 | 2,581,300 (15.0 %) | 10,526,231 (61.4 %) |
| Ishikari Bay, male 3, OE I7 | 12,425,969 | 1,867,374 (15.0 %) | 7,756,367 (62.4 %) |
| Prespawning ground, female 1, OE P2 | 20,751,218 | 3,174,008 (15.3 %) | 12,475,914 (60.1 %) |
| Prespawning ground, female 2, OE P3 | 22,113,503 | 3,370,680 (15.2 %) | 13,305,913 (60.2 %) |
| Prespawning ground, female 3, OE P4 | 26,810,027 | 4,248,086 (15.8 %) | 16,394,714 (61.2 %) |
| Prespawning ground, male 1, OE P7 | 25,914,650 | 4,319,326 (16.7 %) | 15,750,650 (60.8 %) |
| Prespawning ground, male 2, OE P9 | 12,306,800 | 1,901,254 (15.4 %) | 7,227,257 (58.7 %) |
| Prespawning ground, male 3, OE P10 | 12,956,683 | 2,145,609 (16.6 %) | 7,744,958 (59.8 %) |

**Table B: Results of de novo assembly of 12 x PE50 RNAseq datasets.**

| contigs | 98,542 |
| --- | --- |
| Assembly length | 74,391,554 bp |
| N25 | 2,357 bp |
| N50 | 1,254 bp |
| N75 | 520 bp |
| Min length | 200 bp |
| Max length | 17,327 bp |
| Mean length | 755 bp |

**Table C: Bivariate Pearson correlations.** Given are the measured parameters and the Pearson correlation, the significance and the number (N) of individuals for which values were available to perform the analysis for each of the measured parameters. Significance is also indicated by *= P<0.05 and **= P<0.01. Pearson correlation was analysed one-tailed (in the direction of advance of maturation) of log transformed data. Abbreviations: bl= body-length; bw= body-weight; k= condition factor; gsi= gonadosomatic index; hsi= hepatosomatic index; sgnrhfb= *salmon-type gonadotropin-releasing hormone* in the forebrain; sgnrhpb= *sgnrh* in the post brain; gp= *glycoprotein hormone alpha-subunit*; fsh= *fshβ subunit*; lh= *lhβ subunit*; e2= 17β-estradiol; t= testosterone; kt= 11-ketotestosterone; dhp= 17α,20β-dihydroxy-4-pregnen-3-one.

**Table D: The 226 most relevant differentially expressed genes.** Considered as relevant was expression of contigs as based on very stringent criteria: without sasaskin mRNA sequences; expressed in fish of both sites; with p<0.01 and with 2<fc<-2. The expression of 131 genes was up-regulated (green) and expression of 95 genes was down-regulated (red). Shown are the NCBI gene identifier gi; accession number; gene description; P-value and fold change (fc).

| GI version | NCBI ACCESSION | Description | pval | fc |
| --- | --- | --- | --- | --- |
| gi\|209735441 | \|gb\|BT048789.1\| | Salmo salar clone ssal-evd-528-366 CD59 glycoprotein precursor putative mRNA, complete cds | 6.09E-31 | 77.49 |
| gi\|221222033 | \|gb\|BT057806.1\| | Salmo salar clone ssal-rgb2-647-069 Transcription factor HES-5 putative mRNA, complete cds | 2.91E-39 | 20.20 |
| gi\|304376429 | \|gb\|BT046606.2\| | Salmo salar clone ssal-rgb2-504-078 Dexamethasone-induced Ras-related protein 1 precursor putative mRNA, complete cds | 3.58E-12 | 20.12 |
| gi\|470012934 | \|gb\|BT150002.1\| | Salmo salar clone HJ6_0595 metallothionein A mRNA, complete cds | 9.51E-12 | 14.33 |
| gi\|461489474 | \|gb\|JX565544.1\| | Salmo salar insulin-like growth factor binding protein 1 paralog A2 (IGFBP-1A2) mRNA, complete cds | 2.80E-04 | 10.76 |
| gi\|25573081 | \|gb\|AF504024.1\| | Salmo salar MHC class I (UBA) mRNA, UBA*1001 allele, partial cds | 2.29E-18 | 8.35 |
| gi\|209733927 | \|gb\|BT048032.1\| | Salmo salar clone ssal-plnb-027-077 Beta-2-microglobulin precursor putative mRNA, complete cds | 8.16E-03 | 8.13 |
| gi\|209155933 | \|gb\|BT045937.1\| | Salmo salar clone ssal-rgf-537-068 Dehydrogenase/reductase SDR family member 12 putative mRNA, complete cds | 3.36E-04 | 7.39 |
| gi\|221219233 | \|gb\|BT056406.1\| | Salmo salar clone ssal-eve-521-315 3-oxo-5-alpha-steroid 4-dehydrogenase 2 putative mRNA, complete cds | 6.73E-14 | 6.50 |
| gi\|223648117 | \|gb\|BT059104.1\| | Salmo salar clone ssal-rgf-525-310 High mobility group protein B3 putative mRNA, complete cds | 1.01E-10 | 6.20 |
| gi\|350284805 | \|gb\|JN561333.1\| | Salmo salar MHC class I antigen (Sasa-UBA) mRNA, Sasa-UBA*3501 allele, partial cds | 4.55E-15 | 5.95 |
| gi\|221222199 | \|gb\|BT057889.1\| | Salmo salar clone ssal-rgb2-577-292 Hemoglobin subunit beta putative mRNA, complete cds | 3.67E-07 | 5.57 |
| gi\|209733465 | \|gb\|BT047801.1\| | Salmo salar clone ssal-evd-515-074 Metallothionein B putative mRNA, complete cds | 1.51E-18 | 5.57 |
| gi\|304376487 | \|gb\|BT046987.2\| | Salmo salar clone ssal-eve-563-197 Heme oxygenase putative mRNA, complete cds | 6.17E-21 | 5.49 |
| gi\|209731343 | \|gb\|BT046740.1\| | Salmo salar clone ssal-eve-502-285 Tetraspanin-4 putative mRNA, complete cds | 1.33E-03 | 5.42 |
| gi\|221219615 | \|gb\|BT056597.1\| | Salmo salar clone ssal-evd-537-003 Fatty acid-binding protein, brain putative mRNA, complete cds | 8.96E-03 | 5.05 |
| gi\|209734737 | \|gb\|BT048437.1\| | Salmo salar clone ssal-eve-544-124 Mimecan precursor putative mRNA, complete cds | 6.34E-03 | 5.01 |
| gi\|223648739 | \|gb\|BT059415.1\| | Salmo salar clone ssal-rgf-002-184 Procollagen C-endopeptidase enhancer 2 precursor putative mRNA, complete cds | 3.41E-06 | 4.97 |
| gi\|209737803 | \|gb\|BT049970.1\| | Salmo salar clone ssal-eve-572-287 T-cell receptor alpha chain V region 2B4 precursor putative mRNA, complete cds | 8.01E-04 | 4.84 |
| gi\|209154703 | \|gb\|BT045322.1\| | Salmo salar clone ssal-rgf-518-144 Cytochrome P450 4F3 putative mRNA, complete cds | 5.64E-04 | 4.69 |
| gi\|209737253 | \|gb\|BT049695.1\| | Salmo salar clone ssal-evf-521-133 Growth arrest and DNA-damage-inducible protein GADD45 beta putative mRNA, complete cds | 7.38E-05 | 4.66 |
| gi\|221220175 | \|gb\|BT056877.1\| | Salmo salar clone ssal-evf-573-111 Arachidonate 5-lipoxygenase-activating protein putative mRNA, complete cds | 6.05E-04 | 4.53 |
| gi\|221221723 | \|gb\|BT057651.1\| | Salmo salar clone ssal-plnb-503-068 Somatostatin-2 precursor putative mRNA, complete cds | 6.72E-06 | 4.43 |
| gi\|209732639 | \|gb\|BT047388.1\| | Salmo salar clone ssal-evf-523-224 mRNA cap guanine-N7 methyltransferase putative mRNA, complete cds | 9.48E-03 | 4.41 |
| gi\|219809359 | \|gb\|FJ195613.1\| | Salmo salar collagen 2a1 mRNA, partial cds | 9.91E-06 | 4.38 |
| gi\|424012019 | \|emb\|HF543832.1\| | Salmo salar mRNA for beta-carotene 15,15'-monooxygenase 1 (bcmo1 gene) | 1.74E-06 | 4.37 |
| gi\|304376709 | \|gb\|BT048577.2\| | Salmo salar clone ssal-eve-528-247 Plasma retinol-binding protein 1 putative mRNA, complete cds | 4.79E-05 | 4.32 |
| gi\|304376430 | \|gb\|BT046607.2\| | Salmo salar clone ssal-eve-573-286 Leukocyte cell-derived chemotaxin 2 precursor putative mRNA, complete cds | 6.89E-07 | 4.25 |
| gi\|209737699 | \|gb\|BT049918.1\| | Salmo salar clone ssal-eve-539-254 Ubiquitin-like protein precursor putative mRNA, complete cds | 4.32E-03 | 4.24 |
| gi\|198285606 | \|gb\|BT044026.1\| | Salmo salar clone HM6_0837 hypothetical protein mRNA, partial cds | 8.77E-14 | 4.19 |
| gi\|223649253 | \|gb\|BT059672.1\| | Salmo salar clone ssal-rgf-002-035 Ras-related and estrogen-regulated growth inhibitor putative mRNA, complete cds | 6.11E-12 | 4.17 |
| gi\|224587589 | \|gb\|BT072445.1\| | Salmo salar clone ssal-rgf-525-322 Macrophage-expressed gene 1 protein precursor putative mRNA, pseudogene cds | 1.06E-08 | 4.07 |
| gi\|221221583 | \|gb\|BT057581.1\| | Salmo salar clone ssal-eve-566-274 Ubiquitin-like protein 1 putative mRNA, complete cds | 2.60E-05 | 4.04 |
| gi\|226510866 | \|gb\|FJ799990.1\| | Salmo salar matrilin-1 mRNA, partial cds | 1.78E-05 | 3.93 |
| gi\|221221529 | \|gb\|BT057554.1\| | Salmo salar clone ssal-evd-523-297 Ubiquitin-like protein 1 putative mRNA, complete cds | 1.31E-04 | 3.86 |
| gi\|169640207 | \|tpg\|BK006385.1\| | TPA_exp: Salmo salar claudin 5b mRNA, partial cds | 2.90E-03 | 3.85 |
| gi\|221221511 | \|gb\|BT057545.1\| | Salmo salar clone ssal-evd-529-189 Zymogen granule membrane protein 16 precursor putative mRNA, complete cds | 7.59E-07 | 3.81 |
| gi\|209733393 | \|gb\|BT047765.1\| | Salmo salar clone ssal-sjb-012-151 Ubiquitin-like protein 1 putative mRNA, complete cds | 2.29E-04 | 3.73 |
| gi\|209155313 | \|gb\|BT045627.1\| | Salmo salar clone ssal-rgf-526-376 Arrestin domain-containing protein 2 putative mRNA, complete cds | 9.36E-13 | 3.64 |
| gi\|209734219 | \|gb\|BT048178.1\| | Salmo salar clone ssal-evd-523-380 C-C motif chemokine 4 precursor putative mRNA, complete cds | 1.25E-03 | 3.58 |
| gi\|224587259 | \|gb\|BT072122.1\| | Salmo salar clone ssal-rgf-512-199 unknown large open reading frame mRNA, novel cds | 3.86E-10 | 3.58 |
| gi\|469832325 | \|gb\|BT149910.1\| | Salmo salar clone HJ4_3481 cellular retinoic acid-binding protein 1-like protein mRNA, complete cds | 3.09E-06 | 3.58 |
| gi\|221220159 | \|gb\|BT056869.1\| | Salmo salar clone ssal-eve-548-126 Fatty acid-binding protein, brain putative mRNA, complete cds | 1.45E-05 | 3.55 |
| gi\|209731857 | \|gb\|BT046997.1\| | Salmo salar clone ssal-rgb2-626-330 Galectin-9 putative mRNA, complete cds | 2.31E-06 | 3.45 |
| gi\|224613439 | \|gb\|BT072556.1\| | Salmo salar clone ssal-rgf-530-095 Gap junction alpha-1 protein putative mRNA, partial cds | 1.96E-06 | 3.44 |
| gi\|209154967 | \|gb\|BT045454.1\| | Salmo salar clone ssal-rgf-521-374 Tetraspanin-3 putative mRNA, complete cds | 2.14E-04 | 3.40 |
| gi\|221219337 | \|gb\|BT056458.1\| | Salmo salar clone ssal-rgb2-513-269 Eukaryotic translation initiation factor 4E-1A-binding protein putative mRNA, complete cds | 4.88E-07 | 3.38 |
| gi\|209734069 | \|gb\|BT048103.1\| | Salmo salar clone ssal-evf-557-089 Lysozyme g putative mRNA, complete cds | 5.16E-04 | 3.30 |
| gi\|223648433 | \|gb\|BT059262.1\| | Salmo salar clone ssal-rgf-537-204 Transcription factor SOX-9 putative mRNA, complete cds | 6.68E-10 | 3.30 |
| gi\|209154663 | \|gb\|BT045302.1\| | Salmo salar clone ssal-rgf-517-363 SRC-like-adapter putative mRNA, complete cds | 2.89E-03 | 3.26 |
| gi\|221221673 | \|gb\|BT057626.1\| | Salmo salar clone ssal-eve-503-381 Whey acidic protein precursor putative mRNA, complete cds | 3.83E-04 | 3.24 |
| gi\|224613185 | \|gb\|BT071824.1\| | Salmo salar clone ssal-rgf-001-042 Beta-type platelet-derived growth factor receptor precursor putative mRNA, partial cds | 5.06E-03 | 3.22 |
| gi\|209738089 | \|gb\|BT050113.1\| | Salmo salar clone ssal-evf-568-061 Proteasome subunit beta type-8 precursor putative mRNA, complete cds | 8.08E-04 | 3.17 |
| gi\|169640219 | \|tpg\|BK006391.1\| | TPA_exp: Salmo salar claudin 10e mRNA, partial cds | 1.14E-04 | 3.14 |
| gi\|209734549 | \|gb\|BT048343.1\| | Salmo salar clone ssal-evd-548-287 NADH dehydrogenase iron-sulfur protein 5 putative mRNA, complete cds | 1.17E-04 | 3.09 |
| gi\|350284809 | \|gb\|JN561335.1\| | Salmo salar MHC class I antigen (Sasa-UBA) mRNA, Sasa-UBA*3701 allele, partial cds | 1.70E-13 | 3.09 |
| gi\|224587053 | \|gb\|BT071909.1\| | Salmo salar clone ssal-rgf-503-186 unknown large open reading frame mRNA, novel cds | 2.44E-07 | 3.07 |
| gi\|221219751 | \|gb\|BT056665.1\| | Salmo salar clone ssal-eve-510-124 Ependymin precursor putative mRNA, complete cds | 2.19E-04 | 3.00 |
| gi\|221221429 | \|gb\|BT057504.1\| | Salmo salar clone ssal-eve-517-308 Zymogen granule membrane protein 16 precursor putative mRNA, complete cds | 3.72E-08 | 2.93 |
| gi\|224587872 | \|gb\|BT072741.1\| | Salmo salar clone ssal-rgf-538-356 NF-kappa-B inhibitor alpha putative mRNA, pseudogene cds | 1.26E-04 | 2.88 |
| gi\|209734107 | \|gb\|BT048122.1\| | Salmo salar clone ssal-eve-508-057 C-type lectin domain family 4 member E putative mRNA, complete cds | 1.58E-06 | 2.87 |
| gi\|209732665 | \|gb\|BT047401.1\| | Salmo salar clone ssal-evf-531-047 Tissue factor pathway inhibitor 2 precursor putative mRNA, complete cds | 2.23E-07 | 2.80 |
| gi\|221220669 | \|gb\|BT057124.1\| | Salmo salar clone ssal-evd-543-196 Zymogen granule membrane protein 16 precursor putative mRNA, complete cds | 5.85E-14 | 2.79 |
| gi\|223649407 | \|gb\|BT059749.1\| | Salmo salar clone ssal-rgf-503-348 Myeloid-associated differentiation marker putative mRNA, complete cds | 1.03E-05 | 2.78 |
| gi\|223647691 | \|gb\|BT058891.1\| | Salmo salar clone ssal-rgf-511-242 Annexin A11 putative mRNA, complete cds | 8.76E-08 | 2.74 |
| gi\|304376605 | \|gb\|BT047909.2\| | Salmo salar clone ssal-rgb-553-237 Probable protein COQ10, mitochondrial precursor putative mRNA, complete cds | 2.76E-08 | 2.74 |
| gi\|224587224 | \|gb\|BT072087.1\| | Salmo salar clone ssal-rgf-511-001, novel cds | 1.90E-07 | 2.73 |
| gi\|25573077 | \|gb\|AF504022.1\| | Salmo salar MHC class I (UBA) mRNA, UBA*0301 allele, complete cds | 1.86E-06 | 2.73 |
| gi\|221220339 | \|gb\|BT056959.1\| | Salmo salar clone ssal-evd-520-374 Beta-2-microglobulin precursor putative mRNA, complete cds | 1.65E-06 | 2.67 |
| gi\|209735561 | \|gb\|BT048849.1\| | Salmo salar clone ssal-evd-569-162 Lysozyme g putative mRNA, complete cds | 3.17E-05 | 2.67 |
| gi\|209730649 | \|gb\|BT046393.1\| | Salmo salar clone ssal-rgb2-549-178 Testosterone 17-beta-dehydrogenase 3 putative mRNA, complete cds | 8.12E-04 | 2.65 |
| gi\|224613507 | \|gb\|BT072730.1\| | Salmo salar clone ssal-rgf-538-129 Xanthine dehydrogenase/oxidase putative mRNA, partial cds | 8.54E-04 | 2.64 |
| gi\|224587337 | \|gb\|BT072201.1\| | Salmo salar clone ssal-rgf-516-234 unknown large open reading frame mRNA, novel cds | 3.68E-03 | 2.64 |
| gi\|209730499 | \|gb\|BT046318.1\| | Salmo salar clone ssal-evf-504-195 Ankyrin repeat domain-containing protein 57 putative mRNA, complete cds | 2.48E-05 | 2.60 |
| gi\|209733891 | \|gb\|BT048014.1\| | Salmo salar clone ssal-evd-519-176 Ubiquitin-conjugating enzyme E2 D4 putative mRNA, complete cds | 3.73E-03 | 2.58 |
| gi\|221222177 | \|gb\|BT057878.1\| | Salmo salar clone ssal-eve-545-227 Cysteine-rich protein 1 putative mRNA, complete cds | 8.84E-04 | 2.58 |
| gi\|209733723 | \|gb\|BT047930.1\| | Salmo salar clone ssal-evf-570-381 Ras-related C3 botulinum toxin substrate 2 precursor putative mRNA, complete cds | 9.23E-03 | 2.57 |
| gi\|209733689 | \|gb\|BT047913.1\| | Salmo salar clone ssal-eve-006-108 Ferritin, middle subunit putative mRNA, complete cds | 7.70E-03 | 2.57 |
| gi\|221221497 | \|gb\|BT057538.1\| | Salmo salar clone ssal-eve-540-010 15-hydroxyprostaglandin dehydrogenase putative mRNA, complete cds | 1.05E-04 | 2.56 |
| gi\|469832585 | \|gb\|BT150042.1\| | Salmo salar clone FN4_2707 protein-coding gene cg057 like protein mRNA, complete cds | 9.72E-05 | 2.54 |
| gi\|209155505 | \|gb\|BT045723.1\| | Salmo salar clone ssal-rgf-529-273 Cathepsin S precursor putative mRNA, complete cds | 4.83E-04 | 2.54 |
| gi\|224587913 | \|gb\|BT072785.1\| | Salmo salar clone ssal-rgf-540-219 Complement C1s subcomponent precursor putative mRNA, pseudogene cds | 2.83E-04 | 2.51 |
| gi\|221221633 | \|gb\|BT057606.1\| | Salmo salar clone ssal-evf-541-240 atad4-B putative mRNA, complete cds | 2.33E-03 | 2.51 |
| gi\|221221735 | \|gb\|BT057657.1\| | Salmo salar clone ssal-eve-508-073 C3orf68 homolog putative mRNA, complete cds | 4.52E-03 | 2.50 |
| gi\|209730561 | \|gb\|BT046349.1\| | Salmo salar clone ssal-evf-522-118 DnaJ homolog subfamily B member 9 putative mRNA, complete cds | 7.32E-03 | 2.48 |
| gi\|209733231 | \|gb\|BT047684.1\| | Salmo salar clone ssal-eve-515-171 Immunoglobulin superfamily member 6 precursor putative mRNA, complete cds | 2.87E-03 | 2.47 |
| gi\|209154103 | \|gb\|BT045022.1\| | Salmo salar clone ssal-rgf-510-032 Cytochrome P450 1B1 putative mRNA, complete cds | 1.07E-03 | 2.45 |
| gi\|223649495 | \|gb\|BT059793.1\| | Salmo salar clone ssal-rgf-503-059 High mobility group protein B3 putative mRNA, complete cds | 9.38E-06 | 2.45 |
| gi\|209736725 | \|gb\|BT049431.1\| | Salmo salar clone ssal-rgb-553-210 Probable E3 ubiquitin-protein ligase RNF144A-A putative mRNA, complete cds | 1.69E-03 | 2.45 |
| gi\|304269537 | \|gb\|HM452379.1\| | Salmo salar polymeric immunoglobulin receptor-like protein mRNA, complete cds | 6.42E-04 | 2.44 |
| gi\|223649193 | \|gb\|BT059642.1\| | Salmo salar clone ssal-rgf-541-283 Transketolase putative mRNA, complete cds | 3.48E-05 | 2.43 |
| gi\|224613287 | \|gb\|BT072088.1\| | Salmo salar clone ssal-rgf-511-021 Gelsolin precursor putative mRNA, partial cds | 6.24E-05 | 2.43 |
| gi\|224587560 | \|gb\|BT072415.1\| | Salmo salar clone ssal-rgf-524-346 unknown large open reading frame mRNA, novel cds | 1.14E-03 | 2.42 |
| gi\|221221495 | \|gb\|BT057537.1\| | Salmo salar clone ssal-rgb2-547-014 Troponin I, slow skeletal muscle putative mRNA, complete cds | 5.68E-03 | 2.42 |
| gi\|223649331 | \|gb\|BT059711.1\| | Salmo salar clone ssal-rgf-524-184 Integrin beta-1 precursor putative mRNA, complete cds | 9.14E-05 | 2.40 |
| gi\|209155775 | \|gb\|BT045858.1\| | Salmo salar clone ssal-rgf-534-339 NDRG1 putative mRNA, complete cds | 9.63E-07 | 2.39 |
| gi\|224587056 | \|gb\|BT071914.1\| | Salmo salar clone ssal-rgf-503-225 Coagulation factor V precursor putative mRNA, pseudogene cds | 6.88E-03 | 2.36 |
| gi\|260530113 | \|gb\|GQ892057.1\| | Salmo salar polymeric immunoglobulin receptor mRNA, complete cds | 2.73E-03 | 2.36 |
| gi\|198285610 | \|gb\|BT044028.1\| | Salmo salar clone HM6_0880 hypothetical protein mRNA, partial cds | 7.41E-03 | 2.32 |
| gi\|209733623 | \|gb\|BT047880.1\| | Salmo salar clone ssal-evf-572-091 Heme oxygenase putative mRNA, complete cds | 3.85E-03 | 2.30 |
| gi\|223647921 | \|gb\|BT059006.1\| | Salmo salar clone ssal-rgf-519-136 NADPH oxidase 1 putative mRNA, complete cds | 3.93E-03 | 2.28 |
| gi\|223649073 | \|gb\|BT059582.1\| | Salmo salar clone ssal-rgf-516-281 BCL2/adenovirus E1B 19 kDa protein-interacting protein 3 putative mRNA, complete cds | 1.76E-05 | 2.26 |
| gi\|223647517 | \|gb\|BT058804.1\| | Salmo salar clone ssal-rgf-504-050 Glutamine synthetase putative mRNA, complete cds | 1.29E-04 | 2.23 |
| gi\|224587802 | \|gb\|BT072670.1\| | Salmo salar clone ssal-rgf-535-302 Nicotinamide phosphoribosyltransferase putative mRNA, pseudogene cds | 7.54E-03 | 2.20 |
| gi\|25573083 | \|gb\|AF504025.1\| | Salmo salar MHC class I (UBA) mRNA, UBA*0901 allele, partial cds | 1.22E-03 | 2.20 |
| gi\|221221901 | \|gb\|BT057740.1\| | Salmo salar clone ssal-evd-518-359 Acidic leucine-rich nuclear phosphoprotein 32 family member E putative mRNA, complete cds | 8.94E-03 | 2.18 |
| gi\|209733791 | \|gb\|BT047964.1\| | Salmo salar clone ssal-evd-509-248 S100-A1 putative mRNA, complete cds | 6.09E-05 | 2.18 |
| gi\|209155745 | \|gb\|BT045843.1\| | Salmo salar clone ssal-rgf-534-189 SH3 protein expressed in lymphocytes putative mRNA, complete cds | 7.34E-03 | 2.16 |
| gi\|223648875 | \|gb\|BT059483.1\| | Salmo salar clone ssal-rgf-528-103 Branched-chain-amino-acid aminotransferase, cytosolic putative mRNA, complete cds | 4.65E-03 | 2.14 |
| gi\|224587826 | \|gb\|BT072695.1\| | Salmo salar clone ssal-rgf-537-040 Placental protein 11 precursor putative mRNA, pseudogene cds | 8.07E-05 | 2.14 |
| gi\|197632350 | \|gb\|BT043784.1\| | Salmo salar clone HM4_2971 adenylate kinase 1-1 mRNA, complete cds | 1.96E-03 | 2.14 |
| gi\|224613305 | \|gb\|BT072145.1\| | Salmo salar clone ssal-rgf-513-280 Tensin-like C1 domain-containing phosphatase putative mRNA, partial cds | 2.68E-05 | 2.12 |
| gi\|304376662 | \|gb\|BT048301.2\| | Salmo salar clone ssal-rgb2-611-154 Erythrocyte band 7 integral membrane protein putative mRNA, complete cds | 2.08E-04 | 2.11 |
| gi\|221220803 | \|gb\|BT057191.1\| | Salmo salar clone ssal-eve-512-149 Fatty acid-binding protein, brain putative mRNA, complete cds | 6.67E-05 | 2.11 |
| gi\|169640209 | \|tpg\|BK006386.1\| | TPA_exp: Salmo salar claudin 6 mRNA, partial cds | 6.34E-05 | 2.09 |
| gi\|209155101 | \|gb\|BT045521.1\| | Salmo salar clone ssal-rgf-524-002 Cyclin-dependent kinase inhibitor 1B putative mRNA, complete cds | 1.44E-04 | 2.08 |
| gi\|221220363 | \|gb\|BT056971.1\| | Salmo salar clone ssal-rgb2-532-160 Growth arrest and DNA-damage-inducible protein GADD45 gamma putative mRNA, complete cds | 1.79E-03 | 2.08 |
| gi\|224613315 | \|gb\|BT072163.1\| | Salmo salar clone ssal-rgf-514-157 72 kDa type IV collagenase precursor putative mRNA, partial cds | 3.94E-03 | 2.08 |
| gi\|224587556 | \|gb\|BT072410.1\| | Salmo salar clone ssal-rgf-524-264 unknown large open reading frame mRNA, novel cds | 2.86E-04 | 2.07 |
| gi\|378747413 | \|gb\|JN860034.1\| | Salmo salar main olfactory receptor family E subfamily 500 member 1 (MOR500-1) gene, partial cds | 2.95E-03 | 2.06 |
| gi\|223648839 | \|gb\|BT059465.1\| | Salmo salar clone ssal-rgf-513-220 FK506-binding protein 10 precursor putative mRNA, complete cds | 9.50E-04 | 2.06 |
| gi\|209736149 | \|gb\|BT049143.1\| | Salmo salar clone ssal-evf-509-180 C-type lectin domain family 4 member E putative mRNA, complete cds | 1.91E-03 | 2.06 |
| gi\|209153937 | \|gb\|BT044939.1\| | Salmo salar clone ssal-rgf-508-047 Perforin-1 precursor putative mRNA, complete cds | 5.37E-03 | 2.05 |
| gi\|221221891 | \|gb\|BT057735.1\| | Salmo salar clone ssal-evd-552-052 GTPase IMAP family member 7 putative mRNA, complete cds | 9.58E-04 | 2.04 |
| gi\|221221389 | \|gb\|BT057484.1\| | Salmo salar clone ssal-rgb2-616-044 Cytokine-inducible SH2-containing protein putative mRNA, complete cds | 2.07E-04 | 2.04 |
| gi\|304376903 | \|gb\|BT049972.2\| | Salmo salar clone ssal-evd-516-080 Glutathione S-transferase P putative mRNA, complete cds | 3.72E-05 | 2.03 |
| gi\|224587222 | \|gb\|BT072086.1\| | Salmo salar clone ssal-rgf-510-369 Receptor-type tyrosine-protein phosphatase beta precursor putative mRNA, partial cds | 9.53E-05 | 2.03 |
| gi\|223648947 | \|gb\|BT059519.1\| | Salmo salar clone ssal-rgf-512-130 Krueppel-like factor 2 putative mRNA, complete cds | 3.03E-03 | 2.02 |
| gi\|209731027 | \|gb\|BT046582.1\| | Salmo salar clone ssal-evf-534-383 Non-structural maintenance of chromosomes element 1 homolog putative mRNA, complete cds | 8.42E-03 | 2.01 |
| gi\|221221613 | \|gb\|BT057596.1\| | Salmo salar clone ssal-rgb2-549-134 Vacuolar ATP synthase 16 kDa proteolipid subunit putative mRNA, complete cds | 5.83E-04 | 2.01 |
| gi\|209154675 | \|gb\|BT045308.1\| | Salmo salar clone ssal-rgf-518-034 Transcription factor AP-1 putative mRNA, complete cds | 2.07E-03 | 2.00 |
| gi\|223647637 | \|gb\|BT058864.1\| | Salmo salar clone ssal-rgf-508-371 Epithelial cadherin precursor putative mRNA, complete cds | 2.28E-04 | -2.02 |
| gi\|224587002 | \|gb\|BT071859.1\| | Salmo salar clone ssal-rgf-002-348 Collagen alpha-3VI chain precursor putative mRNA, pseudogene cds | 1.12E-03 | -2.03 |
| gi\|198285482 | \|gb\|BT043963.1\| | Salmo salar clone HM5_0922 ribosomal protein S17 (rps17) mRNA, partial cds | 3.71E-03 | -2.04 |
| gi\|209155397 | \|gb\|BT045669.1\| | Salmo salar clone ssal-rgf-528-113 BTG1 putative mRNA, complete cds | 3.70E-05 | -2.04 |
| gi\|224587694 | \|gb\|BT072559.1\| | Salmo salar clone ssal-rgf-530-160 Cellular tumor antigen p53 putative mRNA, partial cds | 1.06E-04 | -2.08 |
| gi\|224587393 | \|gb\|BT072247.1\| | Salmo salar clone ssal-rgf-518-141 CPEB-associated factor Maskin putative mRNA, pseudogene cds | 1.45E-04 | -2.08 |
| gi\|224587386 | \|gb\|BT072240.1\| | Salmo salar clone ssal-rgf-518-031 Mitochondrial glutamate carrier 1 putative mRNA, pseudogene cds | 7.33E-05 | -2.09 |
| gi\|221220181 | \|gb\|BT056880.1\| | Salmo salar clone ssal-evf-522-064 Gastric cancer antigen Zg14 homolog putative mRNA, complete cds | 7.15E-03 | -2.12 |
| gi\|224587145 | \|gb\|BT071999.1\| | Salmo salar clone ssal-rgf-507-021, novel cds | 5.22E-06 | -2.17 |
| gi\|224586999 | \|gb\|BT071856.1\| | Salmo salar clone ssal-rgf-002-227 tRNA selenocysteine-associated protein 1 putative mRNA, pseudogene cds | 5.34E-05 | -2.18 |
| gi\|224587384 | \|gb\|BT072238.1\| | Salmo salar clone ssal-rgf-517-384 unknown large open reading frame mRNA, novel cds | 3.31E-05 | -2.18 |
| gi\|223647763 | \|gb\|BT058927.1\| | Salmo salar clone ssal-rgf-514-032 Arginase-2, mitochondrial precursor putative mRNA, complete cds | 8.57E-05 | -2.18 |
| gi\|221219601 | \|gb\|BT056590.1\| | Salmo salar clone ssal-eve-523-176 regulator of cytokinesis 1 putative mRNA, complete cds | 1.70E-03 | -2.19 |
| gi\|223647497 | \|gb\|BT058794.1\| | Salmo salar clone ssal-rgf-503-184 Serine/threonine-protein kinase Sgk1 putative mRNA, complete cds | 2.47E-04 | -2.19 |
| gi\|197632032 | \|gb\|BT043625.1\| | Salmo salar clone HM4_4021 enoyl Coenzyme A hydratase short chain 1 mitochondrial (echs1) mRNA, complete cds nuclear gene for mitochondrial product | 7.20E-05 | -2.19 |
| gi\|224587847 | \|gb\|BT072715.1\| | Salmo salar clone ssal-rgf-537-273 Arsenite-resistance protein 2 putative mRNA, pseudogene cds | 1.48E-04 | -2.20 |
| gi\|224613383 | \|gb\|BT072403.1\| | Salmo salar clone ssal-rgf-524-139 Mitogen-activated protein kinase kinase kinase 14 putative mRNA, partial cds | 2.42E-05 | -2.22 |
| gi\|209730657 | \|gb\|BT046397.1\| | Salmo salar clone ssal-evf-506-137 60S ribosomal protein L5 putative mRNA, complete cds | 6.84E-05 | -2.23 |
| gi\|223647871 | \|gb\|BT058981.1\| | Salmo salar clone ssal-rgf-517-298 60S ribosomal protein L17 putative mRNA, complete cds | 2.62E-07 | -2.27 |
| gi\|197632090 | \|gb\|BT043654.1\| | Salmo salar clone HM5_1121 40S ribosomal protein S24 (rps24) mRNA, complete cds | 8.04E-03 | -2.29 |
| gi\|221220491 | \|gb\|BT057035.1\| | Salmo salar clone ssal-eve-546-205 Regulator of G-protein signaling 2 putative mRNA, complete cds | 4.01E-05 | -2.30 |
| gi\|221221657 | \|gb\|BT057618.1\| | Salmo salar clone ssal-eve-567-083 Regulator of G-protein signaling 2 putative mRNA, complete cds | 2.60E-04 | -2.30 |
| gi\|304376451 | \|gb\|BT046750.2\| | Salmo salar clone ssal-eve-503-072 Growth arrest and DNA-damage-inducible protein GADD45 alpha putative mRNA, complete cds | 4.12E-04 | -2.30 |
| gi\|209732589 | \|gb\|BT047363.1\| | Salmo salar clone ssal-eve-548-219 Caspase-1 precursor putative mRNA, complete cds | 4.54E-03 | -2.30 |
| gi\|224587198 | \|gb\|BT072057.1\| | Salmo salar clone ssal-rgf-509-218 Sorting nexin-1 putative mRNA, partial cds | 1.97E-06 | -2.33 |
| gi\|223648367 | \|gb\|BT059229.1\| | Salmo salar clone ssal-rgf-535-075 Serine/arginine repetitive matrix protein 1 putative mRNA, complete cds | 5.03E-06 | -2.33 |
| gi\|224587420 | \|gb\|BT072274.1\| | Salmo salar clone ssal-rgf-519-127 unknown large open reading frame mRNA, novel cds | 1.68E-06 | -2.34 |
| gi\|224587816 | \|gb\|BT072684.1\| | Salmo salar clone ssal-rgf-536-203 Transcription factor SOX-9 putative mRNA, partial cds | 1.05E-05 | -2.35 |
| gi\|209731243 | \|gb\|BT046690.1\| | Salmo salar clone ssal-evd-548-033 Thymosin beta-11 putative mRNA, complete cds | 7.31E-03 | -2.36 |
| gi\|224613525 | \|gb\|BT072769.1\| | Salmo salar clone ssal-rgf-539-342 Histone-lysine N-methyltransferase, H3 lysine-9 specific 3 putative mRNA, partial cds | 3.51E-06 | -2.38 |
| gi\|209736989 | \|gb\|BT049563.1\| | Salmo salar clone ssal-evf-522-088 Regulator of G-protein signaling 1 putative mRNA, complete cds | 4.35E-05 | -2.41 |
| gi\|224587435 | \|gb\|BT072285.1\| | Salmo salar clone ssal-rgf-519-307 unknown large open reading frame mRNA, novel cds | 2.62E-05 | -2.43 |
| gi\|209147790 | \|gb\|BT044644.1\| | Salmo salar clone ssal-rgf-001-288 Tubulin alpha chain putative mRNA, complete cds | 1.05E-03 | -2.49 |
| gi\|209733781 | \|gb\|BT047959.1\| | Salmo salar clone ssal-rgb2-587-349 Rieske domain-containing protein putative mRNA, complete cds | 4.18E-03 | -2.52 |
| gi\|224587029 | \|gb\|BT071886.1\| | Salmo salar clone ssal-rgf-502-010 Poly synthetase 1 putative mRNA, partial cds | 3.98E-06 | -2.52 |
| gi\|209732185 | \|gb\|BT047161.1\| | Salmo salar clone ssal-evd-538-229 XTP3-transactivated gene A protein homolog putative mRNA, complete cds | 3.68E-03 | -2.59 |
| gi\|169245904 | \|gb\|EU344852.1\| | Salmo salar Sox9 mRNA, partial cds | 4.32E-03 | -2.60 |
| gi\|223648473 | \|gb\|BT059282.1\| | Salmo salar clone ssal-rgf-538-349 Mediator of RNA polymerase II transcription subunit 15 putative mRNA, complete cds | 5.38E-07 | -2.62 |
| gi\|209734309 | \|gb\|BT048223.1\| | Salmo salar clone ssal-evf-513-381 Histone H3-like centromeric protein A putative mRNA, complete cds | 8.69E-03 | -2.68 |
| gi\|209154473 | \|gb\|BT045207.1\| | Salmo salar clone ssal-rgf-514-311 Insulin-like growth factor-binding protein 6 precursor putative mRNA, complete cds | 1.13E-07 | -2.70 |
| gi\|224587350 | \|gb\|BT072211.1\| | Salmo salar clone ssal-rgf-517-018 Dual specificity protein kinase CLK4 putative mRNA, pseudogene cds | 1.83E-07 | -2.73 |
| gi\|224613443 | \|gb\|BT072561.1\| | Salmo salar clone ssal-rgf-530-188 Condensin complex subunit 3 putative mRNA, partial cds | 1.86E-04 | -2.73 |
| gi\|224613539 | \|gb\|BT072812.1\| | Salmo salar clone ssal-rgf-541-349 Adenylate kinase isoenzyme 2, mitochondrial putative mRNA, partial cds | 3.83E-03 | -2.76 |
| gi\|209154017 | \|gb\|BT044979.1\| | Salmo salar clone ssal-rgf-508-358 Histone chaperone asf1-A putative mRNA, complete cds | 9.46E-06 | -2.77 |
| gi\|209153383 | \|gb\|BT044896.1\| | Salmo salar clone ssal-rgf-506-303 disulfide-isomerase A3 precursor putative mRNA, complete cds | 7.44E-04 | -2.83 |
| gi\|223648879 | \|gb\|BT059485.1\| | Salmo salar clone ssal-rgf-516-265 Cyclic AMP-dependent transcription factor ATF-3 putative mRNA, complete cds | 8.53E-08 | -2.86 |
| gi\|209731925 | \|gb\|BT047031.1\| | Salmo salar clone ssal-eve-536-030 Growth arrest and DNA-damage-inducible protein GADD45 alpha putative mRNA, complete cds | 4.14E-04 | -2.93 |
| gi\|224587245 | \|gb\|BT072109.1\| | Salmo salar clone ssal-rgf-512-018 unknown large open reading frame mRNA, novel cds | 7.83E-05 | -2.93 |
| gi\|221220141 | \|gb\|BT056860.1\| | Salmo salar clone ssal-evd-511-085 Histone H3-like centromeric protein A putative mRNA, complete cds | 1.02E-05 | -2.95 |
| gi\|56967365 | \|gb\|AY848944.1\| | Salmo salar cyclooxygenase 2 (COX-2) mRNA, partial cds | 5.72E-06 | -3.04 |
| gi\|209732607 | \|gb\|BT047372.1\| | Salmo salar clone ssal-eve-530-148 Mediator of RNA polymerase II transcription subunit 15 putative mRNA, complete cds | 1.76E-04 | -3.10 |
| gi\|304376763 | \|gb\|BT048979.2\| | Salmo salar clone ssal-evd-528-084 Nicotinamide riboside kinase 2 putative mRNA, complete cds | 1.62E-03 | -3.17 |
| gi\|221221133 | \|gb\|BT057356.1\| | Salmo salar clone ssal-evd-560-215 Small nuclear ribonucleoprotein F putative mRNA, complete cds | 7.69E-03 | -3.34 |
| gi\|224587540 | \|gb\|BT072391.1\| | Salmo salar clone ssal-rgf-523-235 Rhotekin-2 putative mRNA, pseudogene cds | 2.67E-08 | -3.44 |
| gi\|223647739 | \|gb\|BT058915.1\| | Salmo salar clone ssal-rgf-513-050 Mitotic checkpoint serine/threonine-protein kinase BUB1 putative mRNA, complete cds | 1.25E-07 | -3.51 |
| gi\|224587672 | \|gb\|BT072533.1\| | Salmo salar clone ssal-rgf-529-095 G2/mitotic-specific cyclin-B1 putative mRNA, pseudogene cds | 5.67E-03 | -3.51 |
| gi\|209736091 | \|gb\|BT049114.1\| | Salmo salar clone ssal-eve-557-052 Interleukin-12 subunit beta precursor putative mRNA, complete cds | 7.71E-05 | -3.51 |
| gi\|209730441 | \|gb\|BT046289.1\| | Salmo salar clone ssal-eve-503-094 G2/mitotic-specific cyclin-B1 putative mRNA, complete cds | 5.02E-07 | -3.55 |
| gi\|209155347 | \|gb\|BT045644.1\| | Salmo salar clone ssal-rgf-527-229 BTG2 putative mRNA, complete cds | 1.02E-07 | -3.75 |
| gi\|209731271 | \|gb\|BT046704.1\| | Salmo salar clone ssal-rgb2-650-106 Thymidine kinase, cytosolic putative mRNA, complete cds | 2.49E-05 | -3.78 |
| gi\|224613375 | \|gb\|BT072376.1\| | Salmo salar clone ssal-rgf-523-021 DNA topoisomerase 2-alpha putative mRNA, partial cds | 6.48E-14 | -4.00 |
| gi\|223648557 | \|gb\|BT059324.1\| | Salmo salar clone ssal-rgf-541-174 DNA replication licensing factor mcm5 putative mRNA, complete cds | 1.05E-12 | -4.05 |
| gi\|224587529 | \|gb\|BT072381.1\| | Salmo salar clone ssal-rgf-523-098 DNA replication licensing factor mcm7-A putative mRNA, partial cds | 8.94E-15 | -4.11 |
| gi\|209735385 | \|gb\|BT048761.1\| | Salmo salar clone ssal-evf-547-066 Cornifelin homolog A putative mRNA, complete cds | 1.78E-05 | -4.19 |
| gi\|300067533 | \|gb\|GQ373171.2\| | Salmo salar growth hormone secretagogue receptor 1a variant mRNA, partial cds, alternatively spliced | 3.91E-04 | -4.32 |
| gi\|461489508 | \|gb\|JX565561.1\| | Salmo salar insulin-like growth factor binding protein 6 paralog B2 (IGFBP-6B2) mRNA, complete cds | 9.20E-03 | -4.85 |
| gi\|221219397 | \|gb\|BT056488.1\| | Salmo salar clone ssal-evf-513-150 Growth arrest and DNA-damage-inducible protein GADD45 alpha putative mRNA, complete cds | 3.29E-13 | -4.91 |
| gi\|223648519 | \|gb\|BT059305.1\| | Salmo salar clone ssal-rgf-540-102 Guanine nucleotide-binding protein subunit beta-2-like 1 putative mRNA, complete cds | 3.32E-03 | -4.92 |
| gi\|461489472 | \|gb\|JX565543.1\| | Salmo salar insulin-like growth factor binding protein 1 paralog A1 (IGFBP-1A1) mRNA, complete cds | 3.38E-11 | -4.95 |
| gi\|209731311 | \|gb\|BT046724.1\| | Salmo salar clone ssal-evf-006-290 Serine/threonine-protein kinase SNF1-like kinase 2 putative mRNA, complete cds | 3.43E-09 | -5.05 |
| gi\|224587610 | \|gb\|BT072471.1\| | Salmo salar clone ssal-rgf-526-349 Titin putative mRNA, pseudogene cds | 5.29E-16 | -5.17 |
| gi\|224587709 | \|gb\|BT072572.1\| | Salmo salar clone ssal-rgf-530-323 unknown large open reading frame mRNA, novel cds | 3.23E-13 | -5.29 |
| gi\|221222083 | \|gb\|BT057831.1\| | Salmo salar clone ssal-evd-571-271 Probable thiopurine S-methyltransferase putative mRNA, complete cds | 8.69E-03 | -5.42 |
| gi\|223648845 | \|gb\|BT059468.1\| | Salmo salar clone ssal-rgf-530-199 Fibulin-1 putative mRNA, complete cds | 1.07E-07 | -5.54 |
| gi\|304376444 | \|gb\|BT046699.2\| | Salmo salar clone ssal-evf-503-323 CPEB-associated factor Maskin putative mRNA, complete cds | 3.37E-04 | -5.59 |
| gi\|209731683 | \|gb\|BT046910.1\| | Salmo salar clone ssal-eve-567-293 Granzyme A precursor putative mRNA, complete cds | 1.61E-13 | -5.76 |
| gi\|221220853 | \|gb\|BT057216.1\| | Salmo salar clone ssal-eve-565-357 Growth arrest and DNA-damage-inducible protein GADD45 alpha putative mRNA, complete cds | 4.31E-14 | -6.13 |
| gi\|209732269 | \|gb\|BT047203.1\| | Salmo salar clone ssal-plnb-024-379 Nattectin precursor putative mRNA, complete cds | 8.63E-04 | -6.28 |
| gi\|209735243 | \|gb\|BT048690.1\| | Salmo salar clone ssal-eve-531-073 Granzyme A precursor putative mRNA, complete cds | 2.91E-09 | -6.59 |
| gi\|224587167 | \|gb\|BT072023.1\| | Salmo salar clone ssal-rgf-508-001 Mitochondrial glutamate carrier 1 putative mRNA, pseudogene cds | 1.64E-22 | -6.91 |
| gi\|209735209 | \|gb\|BT048673.1\| | Salmo salar clone ssal-eve-529-313 S100-A1 putative mRNA, complete cds | 5.58E-19 | -7.73 |
| gi\|221219201 | \|gb\|BT056390.1\| | Salmo salar clone ssal-eve-566-101 C-C motif chemokine 20 precursor putative mRNA, complete cds | 4.44E-05 | -7.75 |
| gi\|224613459 | \|gb\|BT072618.1\| | Salmo salar clone ssal-rgf-532-247 Contactin-1a precursor putative mRNA, partial cds | 1.19E-11 | -8.04 |
| gi\|221222231 | \|gb\|BT057905.1\| | Salmo salar clone ssal-evd-565-019 Fatty acid-binding protein, brain putative mRNA, complete cds | 5.02E-06 | -8.42 |
| gi\|221220079 | \|gb\|BT056829.1\| | Salmo salar clone ssal-evf-514-172 Cyclin-dependent kinases regulatory subunit 1 putative mRNA, complete cds | 2.52E-04 | -10.09 |
| gi\|16923150 | \|gb\|AY049952.1\| | Salmo salar vitellogenin (Vtg) mRNA, partial cds | 6.49E-13 | -10.50 |
| gi\|224587433 | \|gb\|BT072284.1\| | Salmo salar clone ssal-rgf-519-300 Serine/threonine-protein kinase Sgk1 putative mRNA, partial cds | 3.42E-33 | -10.91 |
| gi\|261348430 | \|gb\|GU075906.1\| | Salmo salar zona pellucida/egg shell precursor C mRNA, partial cds | 3.52E-04 | -11.20 |
| gi\|221221089 | \|gb\|BT057334.1\| | Salmo salar clone ssal-rgb2-626-139 Fish-egg lectin putative mRNA, complete cds | 3.57E-36 | -12.05 |
| gi\|12408110 | \|gb\|AF327707.1\| | Salmo salar NGFI-B orphan receptor mRNA, partial cds | 5.32E-18 | -12.58 |
| gi\|209156103 | \|gb\|BT046022.1\| | Salmo salar clone ssal-rgf-539-194 Sodium- and chloride-dependent GABA transporter 2 putative mRNA, complete cds | 4.96E-05 | -14.39 |
| gi\|25573061 | \|gb\|AF504014.1\| | Salmo salar MHC class I (UBA) mRNA, UBA*1201 allele, partial cds | 2.10E-09 | -20.78 |
| gi\|209738367 | \|gb\|BT050252.1\| | Salmo salar clone ssal-eve-543-086 Lipocalin precursor putative mRNA, complete cds | 9.53E-25 | -27.44 |
| gi\|213514919 | \|ref\|NM_001141637.1\| | Salmo salar Sporozoite surface protein 2 (ssp2), mRNA | 4.89E-25 | -154.28 |
| gi\|209154623 | \|gb\|BT045282.1\| | Salmo salar clone ssal-rgf-517-144 Sporozoite surface protein 2 precursor putative mRNA, complete cds | 3.86E-48 | -660.05 |

**Table E: The 69 expressed olfactory genes resulting from strategy 1 (mapping reads against 48,223 S. salar NCBI sequences allowing three mismatches).** Expression of 36 of these genes was up-regulated (green) of which for 7 genes significantly (bold). Expression of 33 of the genes was down-regulated (red) of which for 1 gene significantly (bold). Shown are the NCBI gene identifier gi; accession number; gene description; P-value and fold change (fc).

| GI version | NCBI ACCESSION | Description | pval | fc |
| --- | --- | --- | --- | --- |
| gi\|87241878 | \|gb\|DQ375532.1\| | Salmo salar vomeronasal receptor-like protein (SVRA2) gene, partial cds | 0.938 | Inf |
| **gi\|378747413** | **\|gb\|JN860034.1\|** | **Salmo salar main olfactory receptor family E subfamily 500 member 1 (MOR500-1) gene, partial cds** | **0.003** | **2.06** |
| **gi\|378747427** | **\|gb\|JN860041.1\|** | **Salmo salar main olfactory receptor family E subfamily 120 member 2 (MOR120-2) gene, partial cds** | **0.035** | **1.74** |
| **gi\|378747425** | **\|gb\|JN860040.1\|** | **Salmo salar main olfactory receptor family E subfamily 120 member 1 (MOR120-1) gene, partial cds** | **0.016** | **1.74** |
| **gi\|378747417** | **\|gb\|JN860036.1\|** | **Salmo salar main olfactory receptor family E subfamily 500 member 3 (MOR500-3) gene, partial cds** | **0.018** | **1.73** |
| **gi\|378747437** | **\|gb\|JN860046.1\|** | **Salmo salar main olfactory receptor family A subfamily 112 member 1 (MOR112-1) gene, partial cds** | **0.019** | **1.73** |
| gi\|378747447 | \|gb\|JN860051.1\| | Salmo salar main olfactory receptor family F subfamily 115 member 5 (MOR115-5) gene, partial cds | 0.463 | 1.72 |
| gi\|87241891 | \|gb\|DQ375539.1\| | Salmo salar vomeronasal receptor-like protein (SVRC2) gene, partial cds | 0.176 | 1.71 |
| **gi\|378747415** | **\|gb\|JN860035.1\|** | **Salmo salar main olfactory receptor family E subfamily 500 member 2 (MOR500-2) gene, partial cds** | **0.020** | **1.63** |
| gi\|378747401 | \|gb\|JN860028.1\| | Salmo salar main olfactory receptor family E subfamily 121 member 1 (MOR121-1) gene, partial cds | 0.114 | 1.57 |
| **gi\|378747445** | **\|gb\|JN860050.1\|** | **Salmo salar main olfactory receptor family F subfamily 115 member 4 (MOR115-4) gene, complete cds** | **0.032** | **1.53** |
| gi\|224587339 | \|gb\|BT072203.1\| | Salmo salar clone ssal-rgf-516-275 Olfactomedin-4 precursor putative mRNA, pseudogene cds | 0.366 | 1.49 |
| gi\|87241882 | \|gb\|DQ375534.1\| | Salmo salar vomeronasal receptor-like (SVRA4) pseudogene, partial sequence | 0.811 | 1.41 |
| gi\|261206490 | \|gb\|FJ716232.1\| | Salmo salar olfactory receptor family D subfamily 400 member 1 (OR400-1) pseudogene, complete sequence | 0.189 | 1.39 |
| gi\|256274475 | \|gb\|FJ613848.1\| | Salmo salar vomeronasal 1 receptor-like protein (ora1-2) gene, complete cds | 0.154 | 1.38 |
| gi\|378747443 | \|gb\|JN860049.1\| | Salmo salar main olfactory receptor family F subfamily 115 member 3 (MOR115-3) gene, complete cds | 0.229 | 1.35 |
| gi\|329130739 | \|gb\|HM133623.1\| | Salmo salar olfactory receptor family C subfamily 11 member 1 gene, complete cds | 0.108 | 1.34 |
| gi\|378747403 | \|gb\|JN860029.1\| | Salmo salar main olfactory receptor family E subfamily 121 member 2 (MOR121-2) gene, partial cds | 0.344 | 1.34 |
| gi\|378747435 | \|gb\|JN860045.1\| | Salmo salar main olfactory receptor family G subfamily 106 member 3 (MOR106-3) gene, complete cds | 0.315 | 1.31 |
| gi\|329130707 | \|gb\|HM133606.1\| | Salmo salar olfactory receptor family C subfamily 17 member 2 gene, complete cds | 0.149 | 1.30 |
| gi\|378747423 | \|gb\|JN860039.1\| | Salmo salar main olfactory receptor family F subfamily 119 member 1 (MOR119-1) gene, complete cds | 0.128 | 1.30 |
| gi\|87241880 | \|gb\|DQ375533.1\| | Salmo salar vomeronasal receptor-like protein (SVRA3) gene, partial cds | 0.815 | 1.28 |
| gi\|15054055 | \|gb\|AY005457.1\| | Salmo salar olfactory recptor (ORC1) pseudogene, partial sequence | 0.726 | 1.27 |
| gi\|378747405 | \|gb\|JN860030.1\| | Salmo salar main olfactory receptor family E subfamily 121 member 3 (MOR121-3) gene, partial cds | 0.611 | 1.20 |
| gi\|15054058 | \|gb\|AY005460.1\| | Salmo salar olfactory receptor (ORE3) pseudogene, partial sequence | 1.000 | 1.19 |
| gi\|329130710 | \|gb\|HM133608.1\| | Salmo salar olfactory receptor family C subfamily 17 member 3 pseudogene, complete sequence | 0.466 | 1.18 |
| gi\|329130705 | \|gb\|HM133605.1\| | Salmo salar olfactory receptor family C subfamily 17 member 1 gene, complete cds | 0.463 | 1.16 |
| gi\|329130729 | \|gb\|HM133618.1\| | Salmo salar olfactory receptor family C subfamily 12 member 1 gene, complete cds | 0.644 | 1.11 |
| gi\|329130719 | \|gb\|HM133613.1\| | Salmo salar olfactory receptor family C subfamily 16 member 1 gene, complete cds | 0.649 | 1.09 |
| gi\|329130709 | \|gb\|HM133607.1\| | Salmo salar olfactory receptor family C subfamily 17 member p1 pseudogene, complete sequence | 0.575 | 1.07 |
| gi\|378747431 | \|gb\|JN860043.1\| | Salmo salar main olfactory receptor family G subfamily 106 member 1 (MOR106-1) gene, complete cds | 0.636 | 1.07 |
| gi\|378747429 | \|gb\|JN860042.1\| | Salmo salar main olfactory receptor family H subfamily 129 member 1 (MOR129-1) gene, complete cds | 0.951 | 1.02 |
| gi\|378747439 | \|gb\|JN860047.1\| | Salmo salar main olfactory receptor family F subfamily 115 member 1 (MOR115-1) gene, partial cds | 0.557 | 1.02 |
| gi\|329130741 | \|gb\|HM133624.1\| | Salmo salar olfactory receptor family C subfamily 4 member 3 gene, complete cds | 0.921 | 1.01 |
| gi\|329130743 | \|gb\|HM133625.1\| | Salmo salar olfactory receptor family C subfamily 4 member 4 gene, complete cds | 0.869 | 1.01 |
| gi\|329130755 | \|gb\|HM133631.1\| | Salmo salar olfactory receptor family C subfamily 3 member 1 gene, complete cds | 0.852 | 1.00 |
| gi\|15054057 | \|gb\|AY005459.1\| | Salmo salar olfactory receptor (ORE2) pseudogene, partial sequence | 1.000 | -1.01 |
| gi\|329130701 | \|gb\|HM133603.1\| | Salmo salar olfactory receptor family C subfamily 16 member 2 gene, complete cds | 0.976 | -1.02 |
| gi\|329130701 | \|gb\|HM133603.1\| | Salmo salar olfactory receptor family C subfamily 16 member 2 gene, complete cds | 0.976 | -1.02 |
| gi\|329130731 | \|gb\|HM133619.1\| | Salmo salar olfactory receptor family C subfamily 4 member 8 gene, complete cds | 0.768 | -1.05 |
| gi\|378747433 | \|gb\|JN860044.1\| | Salmo salar main olfactory receptor family G subfamily 106 member 2 (MOR106-2) gene, complete cds | 0.811 | -1.05 |
| gi\|329130727 | \|gb\|HM133617.1\| | Salmo salar olfactory receptor family C subfamily 11 member 5 gene, complete cds | 0.893 | -1.05 |
| gi\|329130725 | \|gb\|HM133616.1\| | Salmo salar olfactory receptor family C subfamily 11 member 4 gene, complete cds | 0.924 | -1.05 |
| gi\|329130747 | \|gb\|HM133627.1\| | Salmo salar olfactory receptor family C subfamily 4 member 6 gene, complete cds | 0.687 | -1.07 |
| gi\|329130723 | \|gb\|HM133615.1\| | Salmo salar olfactory receptor family C subfamily 11 member 3 gene, complete cds | 0.696 | -1.09 |
| gi\|329130721 | \|gb\|HM133614.1\| | Salmo salar olfactory receptor family C subfamily 11 member 2 gene, complete cds | 0.618 | -1.10 |
| gi\|378747419 | \|gb\|JN860037.1\| | Salmo salar main olfactory receptor family F subfamily 700 member 1 (MOR700-1) gene, complete cds | 0.454 | -1.10 |
| gi\|329130715 | \|gb\|HM133611.1\| | Salmo salar olfactory receptor family C subfamily 15 member 2 gene, complete cds | 0.747 | -1.10 |
| gi\|329130757 | \|gb\|HM133632.1\| | Salmo salar olfactory receptor family C subfamily 4 member 1 gene, complete cds | 0.540 | -1.10 |
| gi\|329130703 | \|gb\|HM133604.1\| | Salmo salar olfactory receptor family C subfamily 16 member 3 gene, complete cds | 0.532 | -1.13 |
| gi\|329130751 | \|gb\|HM133629.1\| | Salmo salar olfactory receptor family C subfamily 2 member 1 gene, complete cds | 0.395 | -1.16 |
| gi\|329130753 | \|gb\|HM133630.1\| | Salmo salar olfactory receptor family C subfamily 2 member 2 gene, complete cds | 0.463 | -1.16 |
| gi\|329130745 | \|gb\|HM133626.1\| | Salmo salar olfactory receptor family C subfamily 4 member 5 gene, complete cds | 0.432 | -1.16 |
| gi\|329130749 | \|gb\|HM133628.1\| | Salmo salar olfactory receptor family C subfamily 4 member 7 gene, complete cds | 0.424 | -1.17 |
| gi\|329130735 | \|gb\|HM133621.1\| | Salmo salar olfactory receptor family C subfamily 4 member 10 gene, complete cds | 0.419 | -1.21 |
| gi\|256274507 | \|gb\|FJ613864.1\| | Salmo salar olfactory receptor family C subfamily 300 member 3 (salmOR300-3) gene, complete cds | 0.417 | -1.21 |
| gi\|329130713 | \|gb\|HM133610.1\| | Salmo salar olfactory receptor family C subfamily 14 member 1 gene, complete cds | 0.445 | -1.22 |
| gi\|87241887 | \|gb\|DQ375537.1\| | Salmo salar vomeronasal receptor-like protein (SVRB1) gene, partial cds | 0.944 | -1.22 |
| gi\|329130759 | \|gb\|HM133633.1\| | Salmo salar olfactory receptor family C subfamily 4 member 2 gene, complete cds | 0.296 | -1.23 |
| gi\|256274493 | \|gb\|FJ613857.1\| | Salmo salar olfactory receptor family C subfamily 300 member 2 (salmOR300-2) gene, complete cds | 0.498 | -1.24 |
| gi\|329130737 | \|gb\|HM133622.1\| | Salmo salar olfactory receptor family C subfamily 4 member 11 gene, complete cds | 0.136 | -1.29 |
| gi\|329130733 | \|gb\|HM133620.1\| | Salmo salar olfactory receptor family C subfamily 4 member 9 gene, complete cds | 0.347 | -1.31 |
| gi\|256274525 | \|gb\|FJ613873.1\| | Salmo salar olfactory receptor family C subfamily 300 member 4 (salmOR300-4) gene, complete cds | 0.244 | -1.32 |
| gi\|329130717 | \|gb\|HM133612.1\| | Salmo salar olfactory receptor family C subfamily 15 member 1 gene, complete cds | 0.259 | -1.33 |
| **gi\|329130711** | **\|gb\|HM133609.1\|** | **Salmo salar olfactory receptor family C subfamily 13 member 1 gene, complete cds** | **0.045** | **-1.38** |
| gi\|255928902 | \|gb\|FJ611227.1\| | Salmo salar olfactory receptor family C subfamily 300 member 1 (OR300-1) gene, complete cds | 0.104 | -1.44 |
| gi\|87241893 | \|gb\|DQ375540.1\| | Salmo salar vomeronasal receptor-like protein (SVRC3) gene, partial cds | 0.715 | -1.50 |
| gi\|87241883 | \|gb\|DQ375535.1\| | Salmo salar vomeronasal receptor-like protein (SVRA5) gene, partial cds | 0.371 | -1.84 |
| gi\|378747407 | \|gb\|JN860031.1\| | Salmo salar main olfactory receptor family H subfamily 134 member 1 (MOR134-1) gene, complete cds | 0.092 | -1.93 |

**Table F**: **The 43 expressed olfactory genes resulting from strategy 2 (comparing de novo contigs with four reference databases UNIPROT, Teleost Refseq, all Teleost RNAs, *Danio rerio* genome).** Shown are the gene id; the function description; the P-value; the fold change (fc); the result of the salmonid megablast as NCBI gene identifier gi; gene description and the id level. Differentially expressed genes are given in bold.

| **id** | **Function description** | **pval** | **fc** | **megablast 'salmonids'** | **description** | **id level** |
| --- | --- | --- | --- | --- | --- | --- |
| **4676** | **OMP MOUSE Olfactory marker protein** | **0.000** | **2.46** | **gi\|238624075** | **Oncorhynchus nerka sOMP2 mRNA for salmon olfactory marker protein 2, complete cds** | **98%** |
| **38474** | **O52E2 HUMAN Olfactory receptor 52E2** | **0.001** | **2.34** | **gi\|378747413** | **Salmo salar main olfactory receptor family E subfamily 500 member 1 (MOR500-1) gene, partial cds** | **98%** |
| **36765** | **O52K1 HUMAN Olfactory receptor 52K1** | **0.035** | **1.73** | **gi\|185133823** | **Salmo salar odorant receptor ASOR1-like (LOC100136423), mRNA** | **96%** |
| **56146** | **OR5BH HUMAN Olfactory receptor 5B17** | **0.003** | **2.36** | **gi\|378747425** | **Salmo salar main olfactory receptor family E subfamily 120 member 1 (MOR120-1) gene, partial cds** | **96%** |
| **71489** | **O51E2 HUMAN Olfactory receptor 51E2** | **0.005** | **1.93** | **gi\|378747437** | **Salmo salar main olfactory receptor family A subfamily 112 member 1 (MOR112-1) gene, partial cds** | **92%** |
| **13603** | **OLFM4 MOUSE Olfactomedin-4** | **0.027** | **1.75** | **gi\|224587432** | **Salmo salar clone ssal-rgf-519-289 Olfactomedin-4 precursor putative mRNA, pseudogene cds** | **97%** |
| 36035 | O52N5 HUMAN Olfactory receptor 52N5 | 0.061 | 1.75 | gi\|15054055 | Salmo salar olfactory recptor (ORC1) pseudogene, partial sequence | 98% |
| 55521 | OR4E1 HUMAN Olfactory receptor 4E1 | 0.170 | 1.64 | gi\|378747443 | Salmo salar main olfactory receptor family F subfamily 115 member 3 (MOR115-3) gene, complete cds | 96% |
| **17942** | **OLF4 CHICK Olfactory receptor-like protein COR4** | **0.031** | **1.62** | **gi\|378747445** | **Salmo salar main olfactory receptor family F subfamily 115 member 4 (MOR115-4) gene, complete cds** | **86%** |
| 21520 | O11A1 HUMAN Olfactory receptor 11A1 | 0.108 | 1.55 | gi\|378747401 | Salmo salar main olfactory receptor family E subfamily 121 member 1 (MOR121-1) gene, partial cds | 97% |
| 14318 | O5AC2 HUMAN Olfactory receptor 5AC2 | 0.078 | 1.48 | gi\|378747445 | Salmo salar main olfactory receptor family F subfamily 115 member 4 (MOR115-4) gene, complete cds | 97% |
| 47157 | O11A1 HUMAN Olfactory receptor 11A1 | 0.203 | 1.45 | gi\|378747405 | Salmo salar main olfactory receptor family E subfamily 121 member 3 (MOR121-3) gene, partial cds | 76% |
| 11915 | OLFM4 MOUSE Olfactomedin-4 | 0.201 | 1.33 | gi\|224587339 | Salmo salar clone ssal-rgf-516-275 Olfactomedin-4 precursor putative mRNA, pseudogene cds | 97% |
| 4838 | O52J3 HUMAN Olfactory receptor 52J3 | 0.095 | 1.31 | gi\|378747423 | Salmo salar main olfactory receptor family F subfamily 119 member 1 (MOR119-1) gene, complete cds | 96% |
| 5357 | O52N5 HUMAN Olfactory receptor 52N5 | 0.473 | 1.21 | gi\|256274545 | Oncorhynchus gorbuscha olfactory receptor family F subfamily 600 member 1 (salmOR600-1) gene, complete cds | 99% |
| 3627 | O52K1 HUMAN Olfactory receptor 52K1 | 0.379 | 1.20 | gi\|185135609 | Salmo salar main olfactory receptor-like protein (sorf), mRNA | 95% |
| 8039 | OLM2A XENTR Olfactomedin-like protein 2A | 0.502 | 1.17 | gi\|291190193 | Salmo salar Olfactomedin-like protein 2A (olm2a), mRNA | 97% |
| 31071 | OR4D1 HUMAN Olfactory receptor 4D1 | 0.552 | 1.12 | gi\|378747433 | Salmo salar main olfactory receptor family G subfamily 106 member 2 (MOR106-2) gene, complete cds | 95% |
| 17051 | O52D1 HUMAN Olfactory receptor 52D1 | 0.557 | -1.04 | gi\|378747419 | Salmo salar main olfactory receptor family F subfamily 700 member 1 (MOR700-1) gene, complete cds | 98% |
| 16994 | O52N5 HUMAN Olfactory receptor 52N5 | 0.146 | -1.22 | gi\|378747439 | Salmo salar main olfactory receptor family F subfamily 115 member 1 (MOR115-1) gene, partial cds | 96% |
| **21888** | **OLF3A DANRE Olfactomedin-like protein 3A** | **0.017** | **1.86** | **no result** |  |  |
| 44806 | OR2D2 HUMAN Olfactory receptor 2D2 | 0.237 | 1.66 | no result |  |  |
| 34851 | OLM2B HUMAN Olfactomedin-like protein 2B | 0.064 | 1.65 | no result |  |  |
| 56228 | OR5F1 HUMAN Olfactory receptor 5F1 | 0.236 | 1.46 | no result |  |  |
| 23525 | OL142 MOUSE Olfactory receptor 142 | 0.545 | 1.43 | no result |  |  |
| 47099 | O13J1 HUMAN Olfactory receptor 13J1 | 0.352 | 1.39 | no result |  |  |
| 59951 | OR2T5 HUMAN Olfactory receptor 2T5 | 0.515 | 1.29 | no result |  |  |
| 47538 | O11A1 HUMAN Olfactory receptor 11A1 | 0.498 | 1.29 | no result |  |  |
| 13726 | OL226 RAT Olfactory receptor 226 | 0.335 | 1.29 | no result |  |  |
| 3621 | OR4S1 HUMAN Olfactory receptor 4S1 | 0.747 | 1.19 | no result |  |  |
| 59976 | O10G4 HUMAN Olfactory receptor 10G4 | 0.421 | 1.14 | no result |  |  |
| 47655 | O10G7 HUMAN Olfactory receptor 10G7 | 0.830 | 1.13 | no result |  |  |
| 513 | O10G4 HUMAN Olfactory receptor 10G4 | 0.806 | 1.10 | no result |  |  |
| 27449 | O52D1 HUMAN Olfactory receptor 52D1 | 0.950 | 1.09 | no result |  |  |
| 48858 | OR1M1 HUMAN Olfactory receptor 1M1 | 0.452 | 1.07 | no result |  |  |
| 17485 | O52J3 HUMAN Olfactory receptor 52J3 | 0.900 | 1.06 | no result |  |  |
| 30574 | O52D1 HUMAN Olfactory receptor 52D1 | 0.933 | 1.06 | no result |  |  |
| 56711 | O52B2 HUMAN Olfactory receptor 52B2 | 0.810 | -1.04 | no result |  |  |
| 23617 | O1361 RAT Olfactory receptor 1361 | 0.591 | -1.09 | no result |  |  |
| 43688 | O10G7 HUMAN Olfactory receptor 10G7 | 0.973 | -1.10 | no result |  |  |
| 52741 | O10G4 HUMAN Olfactory receptor 10G4 | 0.571 | -1.12 | no result |  |  |
| 47354 | OLFL3 XENTR Olfactomedin-like protein 3 | 0.550 | -1.13 | no result |  |  |
| 53392 | OLM2B HUMAN Olfactomedin-like protein 2B | 0.421 | -1.19 | no result |  |  |
| 1744 | OMP MOUSE Olfactory marker protein | 0.197 | -1.23 | no result |  |  |
| **19342** | **O51F2 HUMAN Olfactory receptor 51F2** | **0.004** | **-1.78** | **no result** |  |  |
| 40015 | CNGA2 HUMAN Cyclic nucleotide-gated olfactory channel | 0.056 | -1.88 | no result |  |  |
| 47355 | OLFL1 HUMAN Olfactomedin-like protein 1 | 0.064 | -4.80 | no result |  |  |
